# Loss of the NF-κB negative regulator Pirk in *Drosophila* links brain and gut immunity to neurodegeneration

**DOI:** 10.1093/braincomms/fcaf144

**Published:** 2025-04-15

**Authors:** Srishti Arora, Grace Critchley, Amira San Dekmak, Gero Miesenböck, Anissa Kempf, Petros Ligoxygakis

**Affiliations:** Department of Biochemistry, University of Oxford, Oxford OX1 3QU, UK; Department of Biochemistry, University of Oxford, Oxford OX1 3QU, UK; Department of Biochemistry, University of Oxford, Oxford OX1 3QU, UK; Centre for Neural Circuits and Behaviour, University of Oxford, OX1 3SR, UK; Centre for Neural Circuits and Behaviour, University of Oxford, OX1 3SR, UK; Department of Biochemistry, University of Oxford, Oxford OX1 3QU, UK

**Keywords:** NF-κB, *Drosophila*, immunity, inflammation, neurodegeneration

## Abstract

A gut–brain axis influenced by host innate immunity and resident microbiota has been implicated in neurological conditions including Alzheimer’s disease. However, the precise connection of innate immunity to Alzheimer’s disease remains unclear. Using Pirk, a negative regulator of the IMD/NF-κB pathway in *Drosophila*, we studied the neurological phenotypes induced when genetically predisposing flies to chronically over-active immunity. *Pirk* mutants exhibited age-dependent neurological phenotypes such as reduced locomotion and altered sleep patterns coupled to an increased number of brain lesions. Gut-specific *pirk-*RNA interference led to earlier onset of the neurological phenotypes which, alongside changes in intestinal bacteria in *pirk* mutants, highlighted a potential early role for the intestinal ecosystem in the onset of neurodegeneration. In contrast, glia-specific RNA interference of *pirk* resulted in late onset of the relevant phenotypes suggesting a later contribution of the nervous system to the underlying neuropathology. Knockout of the antimicrobial peptide (AMP) gene *AttacinD* or rearing flies in axenic conditions recovered some of the neurological phenotypes, suggesting both chronic AMP gene expression as well as gut bacteria changes as mediators. Our results indicate an evolutionarily conserved path to neurodegeneration linked to dysregulated immunity. They also reveal that in this context, age-dependent neurodegeneration can happen in less complex non-vertebrate brains in the absence of beta-amyloid or tau aggregation.

## Teaser

Arora *et al.* explore the connection between chronic inflammation and age-dependent neurodegeneration. In *Drosophila*, flies predisposed to high levels of immunity, neuropathology (such as locomotion defects, sleep pattern alterations and brain lesions) is connected to the gut and intestinal microbiota in young flies and glia-dependent immunity in older adults.

## Graphical Abstract

**Graphical Abstract:**
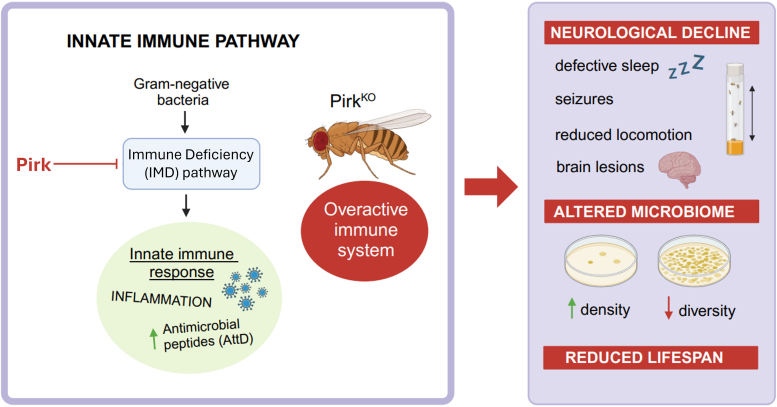

## Introduction

Genome-wide association studies carried out on patients with late onset Alzheimer’s disease as well as in mice expressing β-amyloid, have identified risk genes connected to a brain microglia transcriptional network of innate immune pathways.^1-4^ However, the aetiological connection of these risk genes to the neurological phenotypes observed in late onset Alzheimer’s disease is still an open question. Namely, whether predisposition to aberrant inflammation leads to the hallmark of Alzheimer’s disease (including late onset Alzheimer’s disease), β-amyloid deposition, or if β-amyloid cannot be cleared in certain pro-inflammatory backgrounds.^5^ Nevertheless, there is evidence for the involvement of nuclear factor kappa-light-chain-enhancer of activated B cells (NF-κB) signalling and its downstream targets as part of this inflammatory response.^6^

NF-κB signalling in innate immunity is evolutionarily conserved from flies to humans.^7-9^ Chronic activation of *Drosophila* NF-κB pathway, so called immune deficiency (Imd), leads to age-dependent neurological decline and neurodegenerative disease.^10-13^ Given the connection of immunity to neurodegeneration in humans this indicates an evolutionarily conserved path to neurodegenerative disease. The Imd pathway is activated by bacteria, most strongly by Gram-negative bacteria.^14^ Peptidoglycan in the bacterial cell wall is recognized by the plasma membrane receptor, peptidoglycan recognition receptor LC, which leads to multimerization with subsequent activation of the adaptor protein, Imd.^15^ Fas-associated protein with death domain (dFADD) and death-related Ced-3/Nedd2-like caspase (DREDD) are then recruited by Imd; DREDD (a homologue of caspase 8) cleaves and activates the transcription factor Relish.^16^ The latter is a composite protein containing both an inhibitor of NF-κB (IκB) and NF-κB-like Rel domains. Cleavage by DREDD releases the Rel domain, which can enter the nucleus and regulate target genes. An additional step required for this is phosphorylation by IκB Kinase.^17^ Target genes of Relish include antimicrobial peptide (AMP) genes and genes related to the production of reactive oxygen species. The pathway is negatively regulated at many levels. Details of the components involved, and regulation have been extensively studied and reviewed.^13,18^

This work utilizes mutants of the negative regulator, Poor IMD response upon knock in (Pirk), for predisposing flies to chronic activation of the Imd pathway.^19^ Pirk (otherwise known as Rudra or PIMMS) suppresses the Imd pathway by binding to the cytoplasmic region of peptidoglycan recognition receptor LC with its C-terminal region, hence reducing availability of this receptor at the plasma membrane so that it cannot carry out its normal function in driving Imd signalling.^20^ In the absence of Pirk, the Imd pathway is de-repressed as exemplified by the elevated expression of many AMP genes that are direct transcriptional targets of Relish.^19-21^ *Pirk* is expressed in intestinal stem cells (ISCs).^21^ *Pirk* expression is microbiota dependent, and its presence prevents activation of the IMD pathway against commensal bacteria contributing to homeostasis at the intestinal interphase.^21^ Loss of *pirk* leads to intestinal bacteria changes^21,22^ and a reduction of lifespan.^23^ We have previously shown that loss of *pirk* leads to age-dependent neurological decline, brain lesions and curtailing of lifespan.^11^ In addition, we and others have observed that lifespan of *pirk* mutants was restored to its normal length in axenic conditions.^11,22^ Since *pirk* expression is microbiota dependent, these results open the possibility that the neurodegenerative phenotype observed^11^ was microbiota dependent. This would be in keeping with results implicating key products in the bidirectional relationship between the microbiome and gut in the onset of neurodegeneration in both humans^24^ and flies.^25^ Given the above, we wanted to understand, in the context of *pirk* loss or reduction of function, the contribution of gut and brain immunity in fly age-dependent neurodegeneration.

## Materials and methods

### *Drosophila* stocks and RNAi experiments

*Drosophila* were reared in a 12 h:12 h light:dark cycle at 25°C. Stocks used were obtained from Bloomington *Drosophila* Stock Centre (BDSC) or Vienna stock centre (VDRC) unless otherwise noted: *w^1118^* (BDSC #6326), *yw^67c^*23 (BDSC #6599), *w^1118^; Elav-G4* (BDSC #93288), *w^1118^; Repo-G4* (BDSC #7415), *w^1118^; Esg-G4* (kindly provided by Bruno Lemaitre)57; *w^1118^; Actin-G4* (BDSC #25374), *w^1118^; T(2;3)/Cyo; Δ(2-3), sb* (BDSC #639), *UAS-imd^RNAi^* (VDRC#1284), *UAS-pirk^RNAi^* (VDRC#105790), *yw; pirk^EY00723^* (kindly provided by Bruno Lemaitre),^21^ *w^1118^*; *pirk^EY00723^* (kindly provided by Neil Silverman),^20^ *ΔΑμP10* (kindly provided by Mark Hanson)^26^ and *attD-GFP* strain (kindly provided by Ayano Oi and Fumiaki Obata).^27^ The third chromosome from the *ΔAMP10* stock was used to make the *pirk^EY00723^*; *ΔattD* strain. For knock down of *pirk* and *imd*, we deliberately did not add the temporal *GAL80^ts^* component^28^ in the GAL4/UAS system.^29^ This add-on to the GAL4/UAS system allows for knocking down target genes only in adulthood. However, we wanted to parallel the genetic predisposition to high immune activity of *pirk^ko^*, which does not start in adulthood but is there during development as well.

### Fly food recipe (to 30 l)

Maize flour 1.8 kg, malt extract 1.8 kg, molasses 500 g, soya flour 216 g, yeast powder 366 g, agar (Fisher Scientific, BPE2641-1) 169 g, methyl 4-hydroxybenzoate (Nipagin, Sigma-Aldrich W271004) 74 g, ethanol (VWR) 703 ml, [95% propionic acid (Sigma-Aldrich P5561) 5% phosphoric acid (Sigma-Aldrich P5811) solution 140 ml], water 28 l.

### Generation of *AttacinD-GFP* flies

This has been described elsewhere.^27^

### Excision of pirk^EY00723^

*pirk^EY00723^* flies were crossed with the transposase stock *w^1118^; T(2;3)/Cyo; Δ(2-3), sb* and F1 Cyo/*pirk^EY00723^; Δ(2-3), sb* progeny were crossed with a Cyo/Gla stock. Individual Cyo, non-Gla, and non-sb F1 progeny from this cross were established as single fly crosses backcrossed with Cyo/Gla. Genomic DNA from whole bodies of 15–20 homozygous (non-Cyo) F1 progeny from these single fly crosses was used to assess the presence or absence of a transposable element within the *pirk* gene. Homogenization of flies was carried out using a mechanical rotor and fine needle in β-mercaptoethanol and DNA was extracted using the QIAamp DSP DNA Kit (Qiagen), followed by use of the Nanodrop1000 (Thermo Fisher Scientific) to check its purity and concentration. PCR with MyTaq Red mix (Bioline) was carried out using the T100 Thermal Cycler (Bio-Rad), using conditions stated in the manual. Products were then analysed via gel electrophoresis on a 1.5% agarose gel at 120 V, 400 mA for 45 min, using Hyperladder (Bioline) for quantification of band DNA base pair (bp) size. Primers which were complementary to the long terminal repeat regions of the P{EPgy2} element were utilized in the PCR. Transposable elements are largely composed of long terminal repeat regions, and hence complementary binding of DNA during the PCR reaction and subsequent amplification of DNA products indicate an unsuccessful excision. Primers which bind within 100 bp of the transposable element insertion site were also used; binding is only possible in the absence of a large transposable element and hence they can be used as an additional way to assess whether an excision has been successful.

Primers used: CGACGGGACCACCTTATGTTAT (long terminal repeat), CAGAGACGGAGATAGAGATAGG (Pirk 1 Forward), GCACTCGTCATCGTCATAC (Pirk 1 Reverse), AGCGACAGAGACGGAGATAG (Pirk 2 Forward), AGCACTCGTCATCGTCATAC (Pirk 2 Reverse), GAATGAGCTAACCGACAGAC (Pirk 3 Forward), TTCTATCACACGAACGCCC (Pirk 3 Reverse).

### Rearing of axenic flies

Eggs were first collected with a sterile brush treated with hypochlorite, ethanol, miliQ water and phosphate-buffered saline (PBS) washes. Eggs without eggshells (chorion) were added to autoclaved or antibiotic (tetracycline, penicillin, streptomycin and rifamycin)-treated food. PCR of the 16srRNA gene region and crushing and plating techniques were used to confirm elimination of bacteria in the hatched flies. Control conventionally reared (CR) eggs were treated with miliQ water and PBS and raised in the same conditions as germ-free flies.

### Eosin staining

*Preparation of Eosin stain:* 0.2 g of water-soluble eosin was added to 4 ml of distilled water and mixed until dissolved. Sixteen millilitres of 95% ethanol were added to produce a stock. To make a working solution, 2.5 ml of the stock solution was added to 7.5 ml of 80% ethanol and 50 µl of glacial acetic acid. Eosin stain can be stored at room temperature. *Staining procedure: Drosophila* was placed in 70% ethanol and PBS+ for 30 s, and then 15–20 brains were dissected into PBS+ (0.1% Triton) on ice. They were then fixed with paraformaldehyde (4% in PBS+) for 30 min, followed by three quick and two longer 30-min washes with PBS+ and a 30-min wash with PBS before staining for 2 min in eosin. A working concentration of eosin was produced prior to staining. Eosin was removed, three more quick washes with PBS were carried out, followed by two longer 30-min washes with PBS and a final 15-min wash with PBS+ before leaving over-night rocking at 4°C. All other washes were carried out with rocking at room temperature. Brains were then mounted onto Vector Shield on slides, using three layers of tape between the slide and glass slip to prevent crushing of the brains. Z-stack images were produced using the Olympus FV3000 with a 520–550 nm filter and 20× lens. Quantification of brain lesions was conducted using ImageJ, measuring the accumulated area of lesions across the central region and overall area of the central region at their maximum value in the z-stack, to calculate the % of brain occupied by lesions. Lesions were counted only if they represented a portion of the z-stack rather than damage from top to bottom as this could have been done by handling. For the same reason, we discarded brains that had ‘lesions’ at the periphery as they could have been the result of mis-dissection. *Scoring*: staining and scoring were done by two different experimenters, and both were done blinded (i.e. they did not know the genotype they were staining or scoring).

### Behavioural assays

In all behavioural assays, use of CO_2_ for transfer of flies was avoided to limit potential effects of CO_2_ on behaviour.^30^ All behavioural assays were blinded [i.e. the experimenter did not know which genotype was tested (designated simply 1, 2, 3… by a member of the lab not involved in the study)]. Genotypes were revealed after the analysis of the data.

#### Climbing assay

Locomotor activity of flies was tested with the climbing assay. When a vial of flies is knocked on the bench, they instinctively crawl upwards (negative geotropism). There is evidence of this behaviour being dependent on neurological competency,^31^ making it a very popular behavioural method for assessing neurological decline. As this behaviour is also age dependent, with decline expected during aging, climbing assays are carried out at two time points. Fifteen flies were transferred into 1.5 cm × 14 cm empty plastic vials labelled as three separate quadrants with lines at 3 and 10 cm from the bottom. They were allowed to acclimatize to their new environment for 30 min before testing. Vials were then knocked with the same power three times and the number of flies in each quadrant was counted after 30 s. Three repeats were taken of each vial, with intervals of 5 min in between testing, to account for any differences in experimental technique. The average number of flies in each of the three quadrants was then used to calculate a performance index for each biological replicate: this parameter is often used to represent climbing performance, with 0 representing the worst performance and 1 representing the best. Performance index = 0.5 * (nTotal + nTop − nBottom)/nTotal.

#### Bang-sensitivity assay

Susceptibility to seizures is associated with neurological decline and can be tested using the bang-sensitivity assay.^32-34^ To limit the effect of this assay on other behavioural assays carried out on the same biological replicate of flies, we used a time point distinct from the time points of other assays, and only carried out one technical replicate per genotype. Flies were vortexed in 1.5 cm × 7 cm empty plastic vials for 10 s at maximum speed. Time taken for the flies to recover was recorded; recovery was defined as a fly flipping over and achieving a normal standing position and a recovery time of 1 s reflected a fly that did not appear to have suffered any seizure-like behaviours. To enable easier counting, each biological replicate was transferred into several vials, with only five flies being tested per vial.

#### Sleep assay

Altered sleep activity is another marker associated with neurological and immune effects. *Drosophila* have been shown to have similar sleep patterns to humans.^35^ Flies were placed in glass tubes containing food at one end and a cotton plug at the other. Thirty-two tubes were then loaded into *Drosophila* activity monitors in which an infrared light bisected each tube every minute; a fly was considered active if it cut through the beam, and inactivity for more than 5 continuous minutes was considered sleep. Flies were allowed to acclimatize for a day and then data were collated from the subsequent 2 days. Eight females from each genotype were tested per biological replicate, with three biological replicates in total. Parameters were generated from raw data using the Sleep and Circadian Analysis MATLAB Program, these included frequency of sleep episodes, duration of each sleep episode and total sleep duration. We explored these parameters separately during the day and night to assess overall quality of daytime and night-time sleep.

### Lifespan assays

Flies isogenized over 10 generations were collected on Day 0 and allowed to mate for 2 days. Twenty female flies (3 biological repeats, *n* = 60) were then transferred to a separate vial of food and kept at 25°C. Every 2 days flies were tipped into fresh vials of food, and the number of dead flies was noted. Recorded data were presented as percentage survival over the duration of the maximum lifespan.

### Bacterial density

The protocol was established by adapting crushing and plating techniques used elsewhere.^36^ *Drosophila* were placed in 70% ethanol followed by PBS for 30 s, and guts were dissected into PBS on ice before homogenization using a mechanical rotor and fine needle. Serial dilutions were then performed to reach an appropriate pre-tested dilution in a total volume of 450 μl, which enabled suitable counting of bacterial colony forming units. Two hundred microlitres of homogenized guts were then plated separately onto MRS and LB plates in sterile conditions, using a glass rod sterilized with ethanol and passed through a flame between each plating. Plates were then incubated at 38°C for 72 h. Bacterial colonies were counted using ImageJ; with the Colony Counter plug-in being used where appropriate. Colony forming units were calculated using the following formula: total colony forming units counted on the plate = (dilution factor × amount plated)/total starting volume. Different concentrations of the same homogenized guts and subsequent colony forming unit values were calculated as part of preliminary testing to ensure my technique was sufficient that dilutions were representative of actual colony forming unit values.

### Culture independent gut bacteria identification

Midguts of 40 flies per sample were dissected from 5-day- and 28-day-old male and female flies at selected time points. The dissections were performed in sterile PBS and their genomic DNA (gDNA) was extracted using QIAamp DNA Mini Kit (51304, Qiagen, Germany) according to manufactures instruction along with a reagent control to exclude any contaminants. Genomic DNA was used to amplify the 16S hypervariable regions V1–V3 using Phusion HF DNA polymerase (MO530L, NEB, UK) (PCR conditions). The PCR product was then purified by using the Qiagen PCR Purification Kit (27106, Qiagen, Germany), which was further used to amplify the V3 hypervariable region using Phusion HF polymerase (MO530L, NEB, UK). Finally, the V3 product was run on a 1.2% agarose gel, and a 200 bp V3 band was excised and purified using the QIAquick Gel Extraction Kit (28706, Qiagen, Germany).

The DNA purified from the agarose gel was then measured and approximately 100 ng of V3 DNA samples [measured using Picogreen assay (P11496, Thermo Fisher, USA according to manufacturer’s instructions)] was used for library preparation using NEBNext. Fast DNA Library Prep Set for Ion TorrentTM (E6270, NEB, UK) and Ion XpressTM Barcode Adaptors (4471250, ThermoFisher Scientific, USA). The libraries were further purified using Agencourt AMPure XP DNA purification beads (A63881, Beckman Coulter, Germany) to eliminate any contaminants and primer dimers. Samples were further size selected at 200 bp using a 2% E-gel (G501802, ThermoFisher Scientific, USA) and further quantified using quantitative PCR using KAPA library quantification kits for Ion TorrentTM platform against Ion TorrentTM DNA Standards (KK4812, KAPA Biosystems, USA). Samples that were too low in concentration were re-amplified using the NEBNext. Fast DNA Library Prep Set for Ion TorrentTM (E6270, NEB, UK) protocol and re-quantified. Samples were loaded onto an Ion 314TM Chip v2 (multiplexing 16 samples per chip) using the Ion Chef System and sequenced using the Ion ProtonTM system, (ThermoFisher Scientific, USA) according to manufacturer’s instructions.

ThermoFisher Scientific, USA, provided the Ion ReporterTM software, which was utilized for data analysis. Using V3 primers and a customised metagenomics workflow version 5, the analysis was measured against the microSEQ. 16S reference library v2013.1. Each read was examined for single end primers and reads that were <125 bp (after primer trimming) were disregarded. A minimum alignment coverage of 90% was also applied. Considering the technical inaccuracy of the ion proton, operational taxonomic units were only kept if they had a minimum of three readings, and the species percentage identification was fixed at 99%. It was determined that there was a 0.8% percentage difference between the top hit and the subsequent hit. Graphs were designed using GraphPad Prism version 8. Analytic Rarefaction v1.3 (http://www.uga.edu/∼strata/software/index.html) was used to create rarefaction curves showing sample saturation. PAST3 software was utilized for the examination of beta diversity (principal component analysis) and alpha diversity (Simpson 1-D, Shannon H and Sobs) statistics.

### Analysis of gene expression

Gene expression of mRNA was quantified using quantitative reverse transcription RNA (RT-qPCR). RNA was first extracted from either 15–20 fly whole bodies or 20–30 fly heads using RNeasy kits (Qiagen), and a mechanical rotor and fine needle in β-mercaptoethanol for the initial homogenization step. This step was performed between 12 and 2 p.m. to account for possible circadian rhythm-dependent variation in gene expression. RNA was then subject to DNAse treatment using RNase-Free DNase Set (Qiagen) to remove any contaminating genomic DNA (gDNA). 0.5 μg total RNA was subject to complementary DNA (cDNA) synthesis, using SuperScript III Reverse Transcriptase (ThermoFisher) and following manual conditions. The purity and concentration of RNA and cDNA was assessed at each stage using the Nanodrop1000 (Thermo Fisher Scientific). RT-qPCR of pre-tested cDNA dilutions was carried out using the SensiFAST SYBR No-ROX Kit (Bioline) and the CFX96 Touch Real-Time PCR Detection System (Bio-Rad). Controls which ensured that the generated CT values represented only amplification of the gene of interest include: (i) no template controls containing water in place of a cDNA sample, to ensure there was no gDNA contamination of any of the reagents or contamination caused during the experimental set-up, (ii) no reverse transcriptase controls containing the RNA of each respective cDNA sample in place of cDNA, to ensure there was no gDNA contamination in RNA samples. Relative expression levels were then calculated using the comparative CT method,^37^ normalized against the housekeeping gene, Rp49. Where required, RNA samples were flash frozen using dry ice to prevent degradation.

### Statistical analysis

Statistical analysis and graphs of data were produced using GraphPad Prism, version 9.5.1. Processed sleep, climbing performance and bang-sensitivity data were tested for significance using two-way ANOVA with Tukey’s *post hoc* test (with SEM when error bars were plotted).^38^ Data in S3 were analysed using one-way ANOVA with Tukey’s *post hoc* test (with SEM error bars plotted). Brain lesion comparisons were carried out using the Mann–Whitney test for non-parametric data with Bonferroni correction.^38^ Lifespan data were processed using Kaplan–Meier survival analysis and log-rank tests for comparisons between genotypes. Log10CFU values of mutants and controls were compared using mixed effect analysis to assess bacterial density. RT-qPCR data were compared using unpaired Student’s *t*-tests and Bonferroni correction for multiple comparisons. *P* < 0.05 was considered significant.

## Results

### Loss of *pirk* is associated with neurological decline and neurodegeneration

Human genetics and animal model research have illustrated the aetiological correlation between innate immunity and the pathogenesis of neurodegenerative disorders.^13,39^ However, whether genetic predisposition to chronic innate immune activity can *cause* neurodegeneration is unclear. This is because inflammation can be triggered *consequently* of neuronal death through the production of apoptotic factors and cytokines signalling. To disentangle cause and consequence at the whole animal level, we used *Drosophila* flies predisposed to higher immune activity in the absence of infection.

To achieve this, we used two mutants of *pirk* (Supplementary Fig. 1A). One is a transposable element insertion mutant^21^ with an 82% reduction of *pirk* expression, which we called *pirk* knock out (*pirk^ko^*), while the other an imprecise excision of the transposable element (*pirk^ex1^*) that leads to a partial restoration of expression compared to the genetic background and *pirk^ko^* (Supplementary Fig. 1B). However, the term ‘knock-out’ must be caveated with the fact that it was displaying some expression even when using it over a deficiency, which uncovers the locus (Supplementary Fig. 1B). This suggested that residual mRNA expression was dependent on genomic elements unaffected by the insertion.

**Figure 1:**
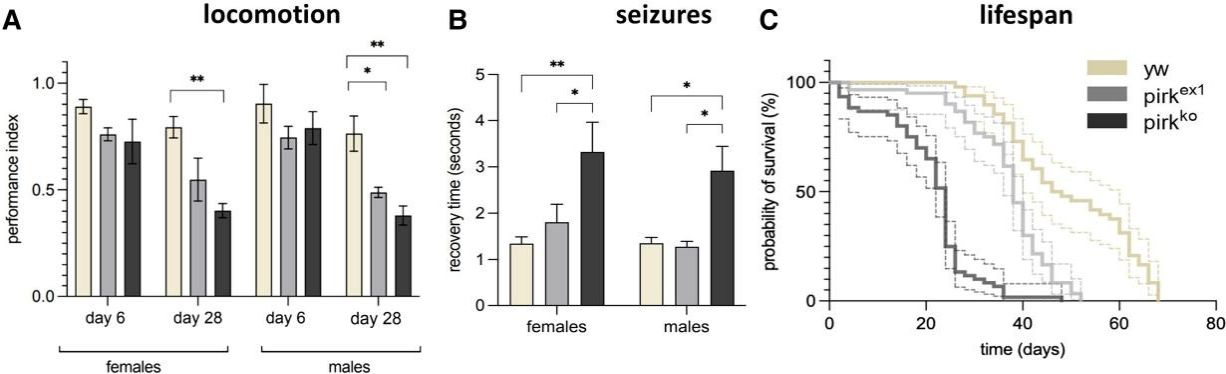
**Behavioural defects and lifespan reduction in isogenic *yw, pirk^ko^* and *pirk^ex1^* mutants. (A)** Locomotion (climbing performance index), of *yw* controls, *pirk^ko^* and *pirk^ex1^* females and males at Days 6 and 28 indicated an age-dependent phenotype. Each bar (*n* = 45) represents the performance index of three biological replicates, each of which is an average of three technical repeats. Statistical analysis by two-way ANOVA, and Tukey’s *post hoc* multiple comparison test (pairwise comparisons plotted on the graph, SEM error bars); *(*P* < 0.05), **(*P* < 0.01). **(B)** Seizure recovery time in *yw* controls, *pirk^ko^* and *pirk^ex1^* female and male flies at Day 16. Each bar (*n* = 45) represents three biological replicates. One-way ANOVA, and Tukey’s *post hoc* multiple comparison test, values shown are mean ± SEM; *(*P* < 0.05), **(*P* < 0.01). **(C)** Kaplan–Meier survival plots (solid lines; 95% CI dotted lines) of isogenic lines of *yw* controls (genetic background), *pirk^ko^* and *pirk^ex1^* females, with each curve (*n* = 90) representing three biological replicates. For comparisons between genotypes, *P*-values were extracted using log-rank tests.

Neurological fitness was tested by assaying propensity to seizures (also called bang-sensitivity), climbing ability (as a proxy for locomotion) and sleep patterns. Our results corroborated the age-dependent reduction of locomotion described previously,^11^ which we now report separately for females and males (Fig. 1A). We also observed an increase in the susceptibility to seizures in both sexes (Fig. 1B). As shown previously^11,22^ *pirk^ko^* had a significantly reduced lifespan (LT_50_ = 23 days) compared to its *yw* genetic background (LT_50_ = 48 days) (Fig. 1C). Neurological tests showed that *pirk^ex1^* flies had climbing ability (Fig. 1A) and susceptibility to seizures (Fig. 1B) that was between the *yw* genetic background and *pirk^ko^*. This was also the case for their lifespan (Fig. 1C). This indicated that *pirk* phenotypes were dose-dependent, correlating with the levels of *pirk* mRNA (Supplementary Fig. 1B).

However, with regards to seizure-like behaviour, we note that recovery of bang-sensitive mutants is divided into two categories: ‘mild’ (average recovery time of <30 s) and ‘strong’ (requiring >30 s).^32-34^ Here, *pirk^ko^* and *pirk^ex1^* mutants took an average of 5–6 s (versus 1 s in controls) to recover. We therefore hypothesized that instead of seeing a true propensity to seizures (with tonic-clonic-like movements),^40^ we were corroborating the locomotion defects detected in the climbing assays. Therefore, we excluded seizure assays from the rest of our investigation. From the two *pirk* alleles, we henceforth focus on *pirk^ko^*.

Defective sleep is associated with many different neurodegenerative diseases and is used as a biomarker for late onset Alzheimer’s disease, as disturbances in sleep appear long before any cognitive decline is apparent.^41^ Moreover, individuals with defective sleep were shown to have a 3.78 times higher likelihood of developing Alzheimer’s disease.^42^ Whether defective sleep drives neurodegeneration in patients or is a consequence that arises from loss of neural cells involved in sleep regulation is still debated.^43^ Sleep is also heavily intertwined with immunity in mammals^44^ as well as *Drosophila.*^45,46^

After examining the effects of *pirk^ko^* on climbing, we tested whether *pirk^ko^* mutants aged 6 and 28 days old, exhibited changes in sleep and activity behaviour and whether any effect was exacerbated with age. All flies used were free of Wolbachia as this bacterial endosymbiont influences sleep.^47^ To mitigate for genetic background effects, we incorporated *pirk^ko^* in a genetic background different than *yw* namely, the *w^1118^* stock. As anticipated, both *pirk^ko^* and *w^1118^* female flies had predictable circadian rhythm (Supplementary Fig. 2A). Female *w^1118^* flies showed more activity during the light phase (12 h duration, 9 a.m.–9 p.m.) than dark (12 h duration, 9 p.m.–9 a.m.). In those *w^1118^* flies an age-dependent reduction of sleep was observed during the light phase. In contrast, *pirk^ko^* female flies slept more at both 6-day- and 28-day-old time points compared to their genetic background, with the 28-day time point showing the highest amount of daytime sleep (Supplementary Fig. 2A).

Like female flies, male *w^1118^* flies displayed a predictable circadian rhythm (Supplementary Fig. 2B). However, unlike female flies, *w^1118^* males seemed to sleep more during the day at both 6-day and 28-day time points. Nevertheless, we still observed an age-dependent increase in daytime sleep in both *w^1118^* and *pirk^ko^* males. Overall, we could deduce that older *pirk^ko^* males may be sleeping more during the day than controls. However, this needed to be confirmed by other sleep profiles. For this, we divided total sleep (and all other parameters) into daytime (12 h duration, lights on) and night-time (12 h duration, lights off).

**Figure 2:**
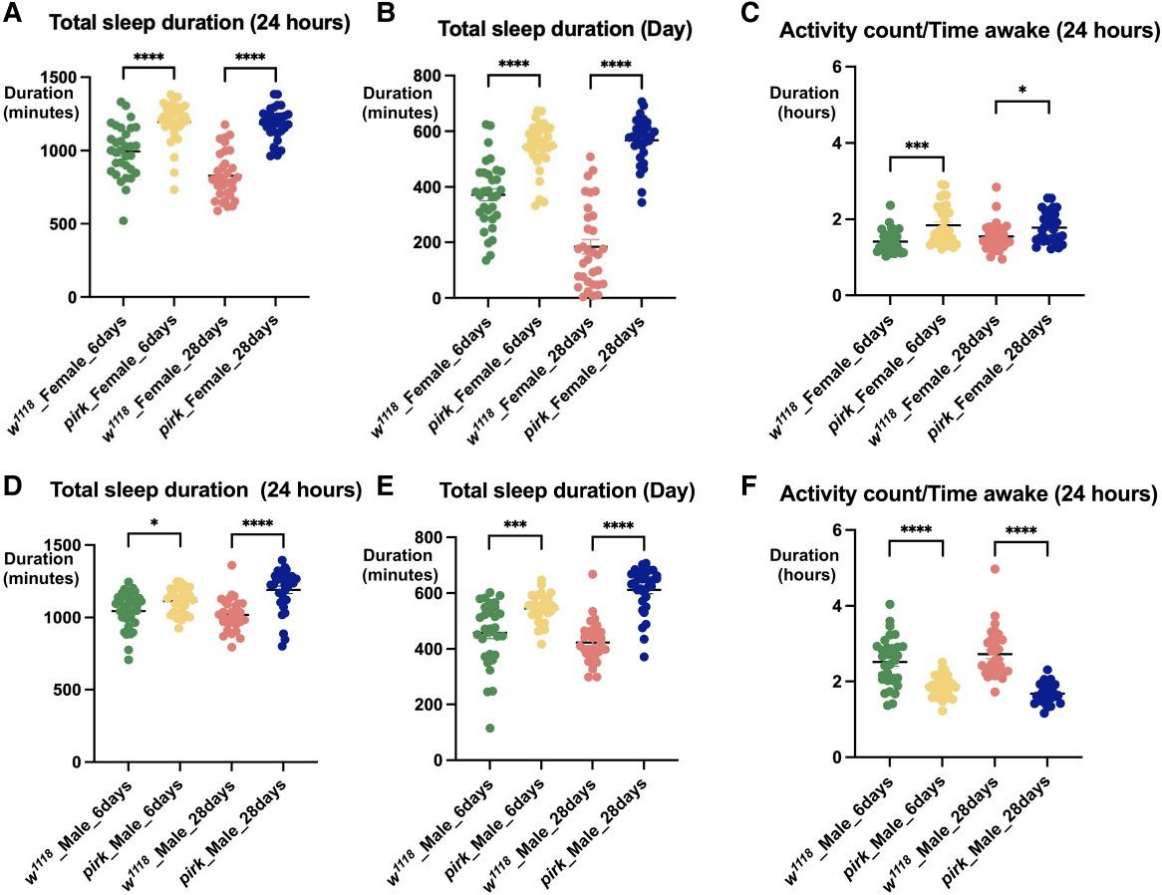
**The effect of *pirk^ko^* mutation on sleep profiles of 6 and 28-day-old flies. (A)** Total (24 h) sleep (females) **(B)** Total (day) sleep (females) **(C)** Activity when awake (females) **(D)** Total (24 h) sleep (males) **(E)** Total (day) sleep (males) **(F)** Activity when awake (males). Experiments were performed with 32 flies per genotype per sex per time point in three biological replicates (total *n* = 96 per genotype per sex and per time point). Data were evaluated for following normal distribution and analysed by performing a two-way ANOVA, and Tukey’s *post hoc* multiple comparison test, and *P*-values were extracted. A *P* < 0.05 was considered statistically significant, bars represent SEMs (****P < 0.0001, **P < 0.01, *P < 0.05). Each data point represents an individual fly.

Total sleep duration is calculated as total minutes of sleep during a 24 h period. We observed that female *pirk^ko^* flies had a significant increase in total sleep duration at both 6 and 28 days (*P* < 0.0001, n = 32 × 3 biological repeats = 96) (Fig. 2A). The median sleep of 6- and 28-day *pirk* females were 1226 and 1192 min, respectively as opposed to 1002 and 806.5 min for 6 and 28 days *w^1118^* female controls (Fig. 2A).

Total sleep per 24 h can further be divided into total duration of sleep during the day (light phase) and total duration of sleep at night (dark phase). Like total sleep duration, we observed a significant rise in total sleep during the day in *pirk^ko^* females at both 6- and 28-daytime point compared to their respective controls (*P* < 0.0001, n = 96) (Fig. 2B). This indicated that *pirk^ko^* females were sleeping significantly more during the day. When awake, *pirk^ko^* females were more active than controls in both time points indicating a specific effect of *pirk^ko^* on sleep rather than a general effect of frailty (Fig. 2C).

We observed a significant increase in the total (24 h) duration of sleep in both 6-day (*P* = 0.0258, *n* = 32) and 28-day-old (*P* < 0.0001, *n* = 32) male *pirk^ko^* flies with a median increase in sleep of 62 min in younger flies and an increase of 220 min in older ones (Fig. 2D). This was connected to a substantial increase in day sleep observed in both 6-day (*P* = 0.0006, *n* = 32) and 28-day (*P* < 0.0001, *n* = 32) old male *pirk^ko^* mutants compared to their *w^1118^* controls (Fig. 2E). In contrast to female *pirk^ko^* flies, *pirk^ko^* males were less active when awake than controls from early on (6-day-old flies) (Fig. 2F). In conclusion, we observed an age-dependent increase in sleep of *pirk^ko^* flies, especially during the day.

To consolidate the behavioural phenotypes associated with the *pirk* mutants we compared the climbing behaviour of the *pirk^ko^* allele with ubiquitous *pirk* knockdown *via* RNAi in both females and males. To this end, we used the GAL4/UAS system^38^ to knock-down *pirk* with an *actinGAL4* driver. In both sexes, ubiquitous knock-down of *pirk* was statistically indistinguishable from *pirk^ko^* and reproduced the age-dependent decline in locomotion of *pirk^ko^* flies from 6-day-old (Supplementary Fig. 3A) to 28-day-old (Supplementary Fig. 3B) compared to both genetic backgrounds (*yw* and *w^1118^*).

Taken together, the above data showed that near loss or reduction of *pirk* expression resulted in early symptoms of neurological decline. These symptoms have been correlated with neurodegeneration. We next asked whether the constitutive expression of immune effectors (such as IMD-dependent AMPs) in *pirk* mutants was contributing to the observed neurological decline and looked for evidence of brain lesions.

### AttacinD is an immune effector linking immunity and neurodegeneration in *pirk* mutants

AMPs have been associated with neurodegeneration in mammals, *e.g*. the AMP LL-37 which stimulates glial cells to release pro-inflammatory cytokines and chemokines in Alzheimer’s disease progression.^48^ In the *Drosophila* brain, transgenic overexpression of AMPs results in neurodegeneration.^10^ Moreover, our previous studies have shown that expression of Imd-dependent AMPs in the brain increases in an age-dependent manner.^11^ In this context, AttacinD (AttD) is highly expressed in *pirk^ko^* flies (Supplementary Fig. 4). Making use of the *ΔAMP10* fly stock developed in the Lemaitre lab^26^ where most *Drosophila* AMP families are deleted, we combined *pirk^ko^* with the third chromosome of the *ΔAMP10* fly stock that includes the deletion of *AttD*. Of note, reduced climbing has been observed in *ΔAMP10.*^49^

**Figure 3:**
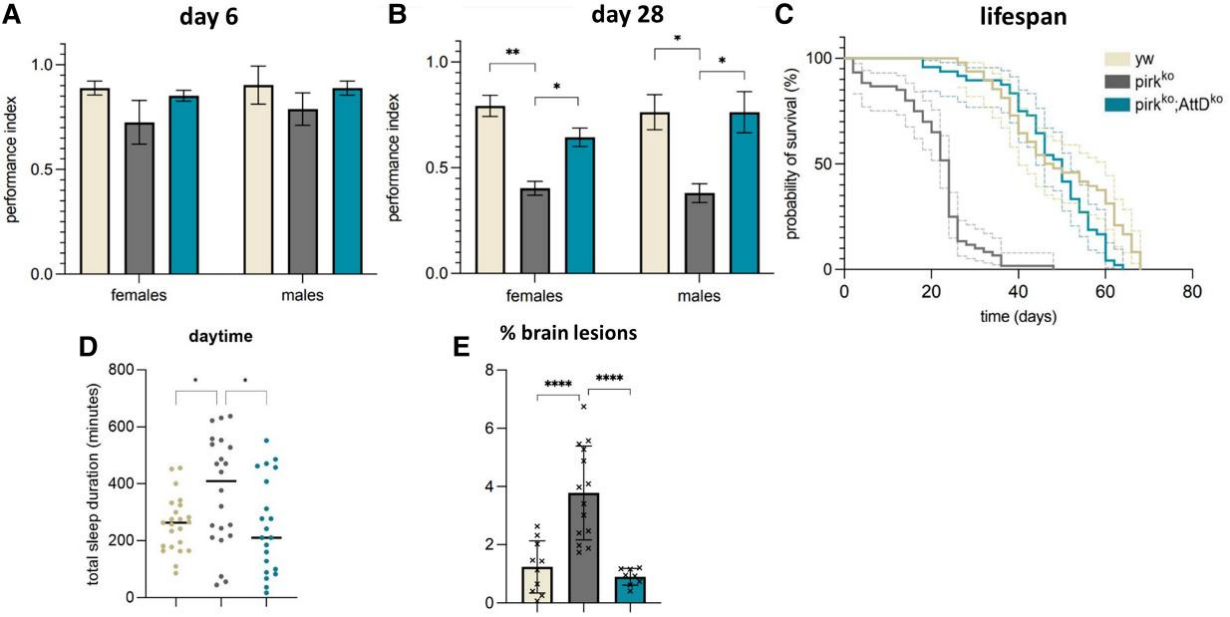
**Knockout of *AttD* in the context of *pirk^ko^* rescues neurological defects and lifespan. (A)** Locomotion (climbing performance index) for isogenic *yw* controls, *pirk^ko^* and *pirk^ko^*; *AttD^ko^* female and male flies at Day 6 and **(B)** at Day 28. Each bar represents three biological replicates, each of which is an average of three technical repeats (*n* = 45). Values shown are mean ± SEM, using two-way ANOVA with Tukey’s *post hoc* analysis test (*P < 0.05, **P < 0.01). **(C)** Survival curves of isogenic *yw* controls, *pirk^ko^* and *pirk^ko^*; *AttD^ko^* female flies. Each curve represents three biological replicates (*n* = 90 per genotype; Kaplan–Meier survival plots and log-rank tests for comparisons between genotypes; solid lines; 95% CI dotted lines). **(D)** Total duration of sleep during the day in Day 6. **(E)** % percentage of whole brains occupied by lesions in *yw* controls, *pirk^ko^* and *pirk^ko^; AttD^ko^* females at Day 28. Values shown are mean ± SEM with ANOVA and Tukey’s *post hoc* test (****P < 0.0001).

*Pirk^ko^* mutants with a deletion of *AttD* showed significant improvement across all locomotion assays compared to *pirk^ko^* mutants, with substantial evidence of similar behaviour to *yw* controls (Fig. 3). There was a significant improvement of climbing defects in both females and males at 28 days (Fig. 3B). Moreover, lifespan was fully recovered in female *pirk^ko^*; *AttD^ko^* double mutants compared to the *yw* controls, with a median survival age of 50 days compared to 47 days for the *yw* control (*P* = 0.0263) and 24 days for *pirk^ko^* mutants (*P* < 0.0001) (Fig. 3C).

*AttD* knockout in *pirk^ko^* mutants restored their sleep patterns. At Day 6, double *pirk^ko^; AttD^ko^* knockout mutants showed the increase in daytime sleep associated with *pirk^ko^*, reduced back to control levels (Fig. 3D). Next, we wanted to correlate the neurological phenotypes with brain histology. Eosin was chosen as an appropriate stain for simple quantification of neurodegeneration. Figure 3E shows that *pirk^ko^* mutants had a significantly higher percentage of their central brain occupied by lesions compared to *yw* control while *pirk^ko^; AttD^ko^* mutants were indistinguishable from controls (for brain images, see Supplementary Fig. 5). This finding supports data from our behavioural assays, indicating that *AttD* knockout in *pirk^ko^* mutants helped to recover climbing ability and sleep patterns.

### Pirk knockdown in intestinal stem cells or the nervous system contributes to the observed neurological phenotypes

**Figure 4:**
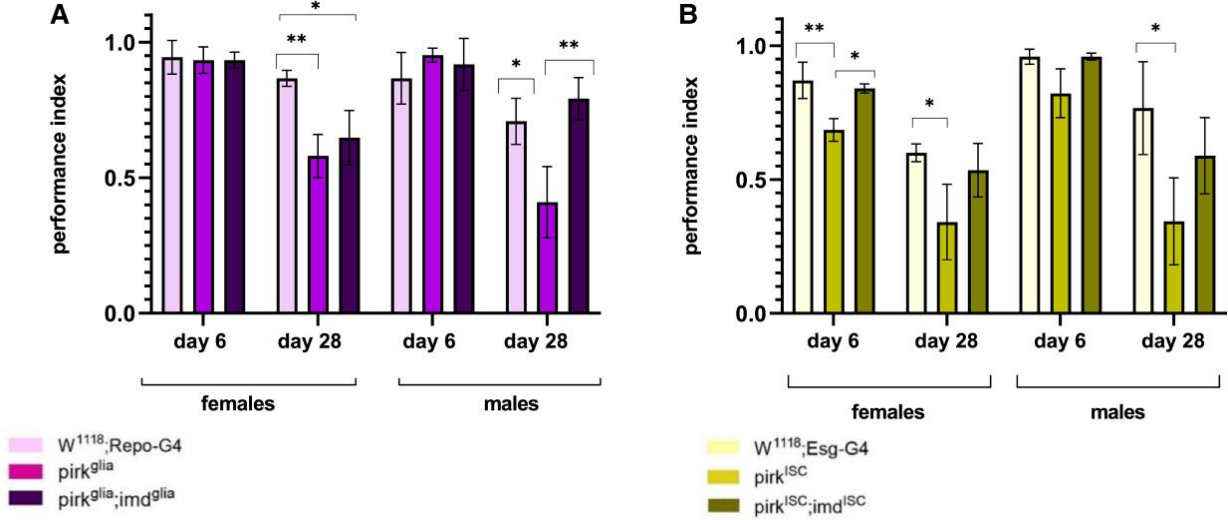
**Imd knockdown rescued many of the significant locomotion defects seen in pirk^glia^ and pirk^ISC^ RNAi. (A)** Locomotion of *w^1118^; Repo-GAL4, UAS-Pirk^RNAi^*; *UAS-mCherry^RNAi^*/Repo-G4 and *UAS-Pirk^RNAi^; Repo-G4/UAS-Imd^RNAi^* flies. **(B)** Locomotion of *w^1118^; esg-G4, esg-G4/UAS-Pirk^RNAi^; UAS-mCherry^RNAi^* and *esg-G4/UAS-Pirk^RNAi^;UAS-Imd^RNAi^* flies. Locomotion is represented by performance index, each bar represents three biological replicates, each of which is an average of three technical repeats (*n* = 45, per genotype per sex per time point). Climbing is compared at Day 6 and Day 28. Values shown are mean ± SEM. *P < 0.05, **P < 0.01, ***P < 0.001, ****P < 0.0001 (two-way ANOVA with Tukey’s *post hoc* analysis test).

To ascertain the contributions of the gut (where *pirk* is expressed in ISCs^21^) and the nervous system to the *pirk^ko^* phenotypes, we proceeded to silence *pirk* in these tissues. RNAi-mediated knockdown of *pirk* in different tissues recapitulated the *pirk^ko^* neurological phenotype to varying degrees. With regards to climbing performance (locomotion), the earliest onset of defects (Day 6) occurred with *pirk* knockdown in Intestinal Stem Cells (ISCs) (*pirk^ISC^*) of female flies (Fig. 4A). In contrast, knock down in glia (*pirk^glia^*) or neurons (*pirk^neuro^*) had no early impact on locomotion in either sex (Fig. 4A, Supplementary Fig. 6). At Day 28, *pirk^glia^* and *pirk^ISC^* female flies showed a significant reduction in their climbing ability. No significant differences were seen for *pirkneuro* in 28-day female flies (Supplementary Fig. 6). In 28-day-old males however, all RNAi lines showed significant reductions relative to controls (Fig. 4B, Supplementary Fig. 6). Taken together, these results showed that the age-dependent effects on locomotion seen in *pirk^ko^* males, were reproduced by all driver lines tested, while only by *pirk^ISC^* and *pirk^glia^* in females.

**Figure 5:**
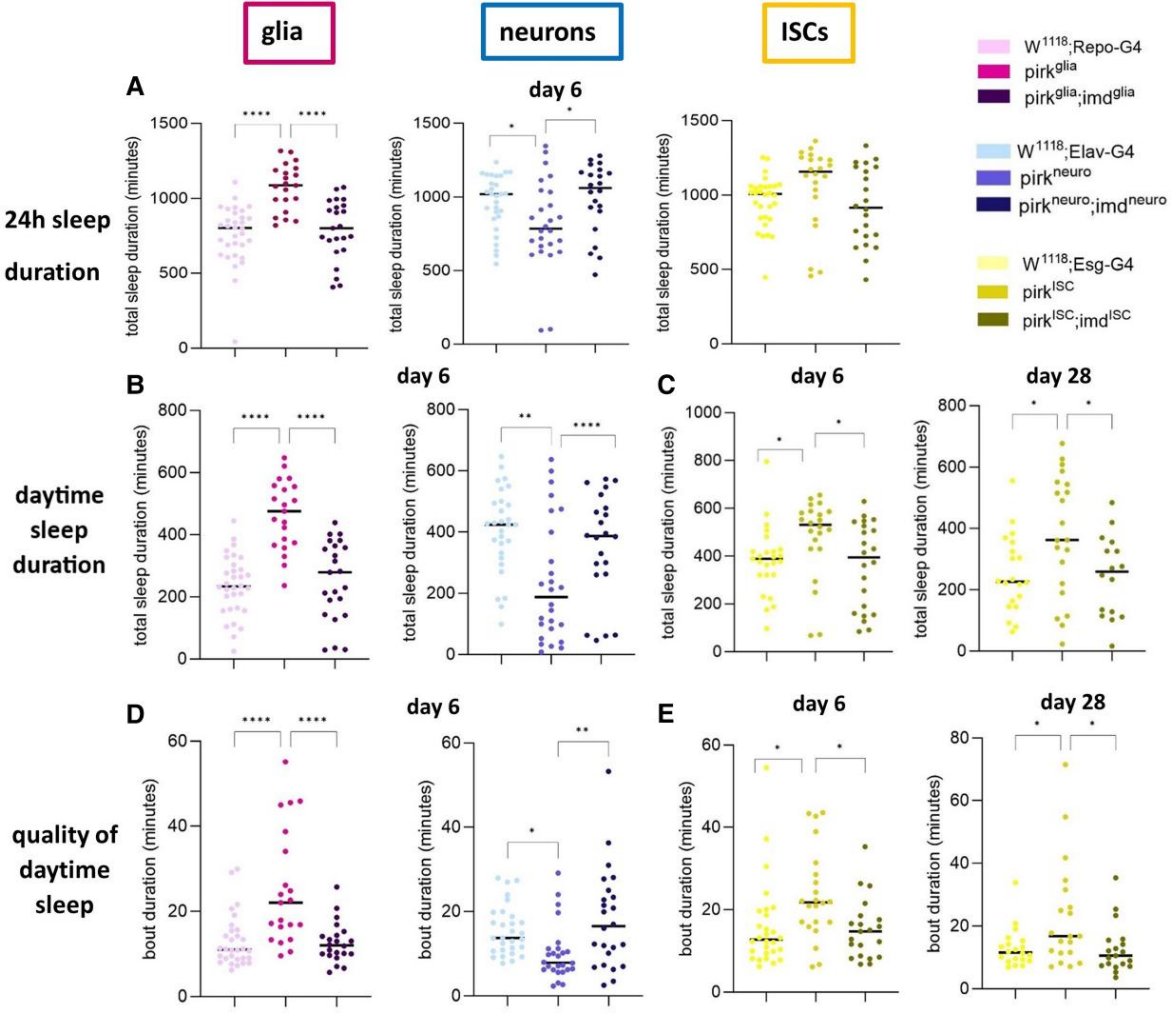
**Rescue of daytime sleep defects in *pirk^glia^*, *pirk^neuro^* and *pirk^ISC^* mutants by concomitant *imd* knockdown. (A)** Total sleep duration over 24 h is compared at Day 6 across all tissue [glia, neurons, intestinal stem cells (ISCs)]-specific RNAi. **(B)** Total daytime sleep is compared at Day 6 in glia and neuron-specific RNAi. **(C)** Sleep bout durations compared in ISC-specific RNAi at Day 6 and Day 28. **(D)** Sleep bout durations compared at Day 6 in glia and neuron-specific *pirk^glia^* RNAi. **(E)** Sleep bout durations at Day 28 in ISC-specific RNAi. All panels represent three biological replicates (*n* = 96 per genotype, per time point) with two-way ANOVA and Tukey’s *post hoc* test (*P < 0.05, **P < 0.01, ****P < 0.0001), each data point denotes a single female fly.

Relative to controls, total (24 h) sleep duration was significantly decreased in Day 6 *pirk^neuro^* and elevated in Day 6 *pirk^glia^* while not significantly changed in *pirk^ISC^* (Fig. 5A). No statistically significant change in 24 h sleep was observed at Day 28 for any RNAi treatment (Supplementary Fig. 7). However, even with no net 24 h sleep changes, equivalent shifts between day and night-time sleep could still occur. Therefore, we looked at day and night sleep separately. Agreeing with their 24-h patterns at Day 6, daytime sleep was affected in *pirkneuro* (decreased) and *pirkglia* (increased) (Fig. 5B). Additionally, we observed an increase of daytime sleep of *pirk^ISC^* in both 6- and 28-day-old flies (Fig. 5C). When compared to *pirk^ko^* therefore, only *pirk^ISC^* reproduced the increase in total daytime sleep across the life-course. We next checked the quality of sleep, which was assessed by looking at the frequency and duration of sleep episodes (bouts). These parameters could potentially explain the changes seen in daytime sleep. Elevated daytime sleep, in Day 6 *pirk^glia^* mutants, appeared to be mediated by an increase in the length of each sleep episode (Fig. 5D). This was also seen in *pirk^ISC^* in Day 6 and Day 28 (Fig. 5E). Reduction of daytime sleep in *pirk^neuro^* appeared to occur *via* a reduction of the number of sleep episodes (shown at Day 6; no significant differences were seen at Day 28 see Supplementary Fig. 7). Moreover, RNAi of *pirk* in the nervous system increased night-time sleep through an increase in duration of sleep bouts of 6-day but not 28-day-old *pirk^glia^* (Supplementary Fig. 8A) or *pirk^neuro^* (Supplementary Fig. 8B).

Taken together these results showed that increases in sleep were due to longer sleep bout durations, while decreases in sleep were due to a reduction in the number of sleep bouts. Moreover, the phenotype of significant increase in daytime sleep in *pirk^ko^* was matched by *pirk^ISC^* (across the life course) and *pirk^glia^* (Day 6) but not with RNAi of *pirk* in neurons.

### Imd knockdown can rescue the neurological phenotypes caused by *pirk* knockdown

To attribute the effects of *pirk* knockdown mutants to tissue-specific overactivation of the Imd pathway thus linking this immune pathway to neurological decline, we concomitantly knocked down *imd* in the same tissue as *pirk*. As *pirk* knockdown leads to overactivation of the Imd pathway, *imd* knockdown should rescue at least some of the defects. However, to balance the UAS sites available for GAL4 in *pirk* single and *pirk*; *imd* double knockdown we used as control a *UAS-pirk^RNAi^*; *UAS-mCherry^RNAi^* stock to compare to the *UAS-pirk^RNAi^*; *UAS-imd^RNAi^*. Concomitant RNAi of *imd* and *pirk* in glia did not rescue climbing defects infemales at Day 28 (Fig. 4A), but did recover climbing defects in females of *pirk^ISC^* at Day 6 (Fig. 4B). Although there was less evidence of reduced climbing performance of *pirk^neuro^* mutants relative to the GAL4 control (only 28-day-old *pirk^neuro^* males), it is still useful to consider the effect of *Imd* knockdown here. *Pirk^neuro^ imd^neuro^* double knockdown led to significantly better locomotion than *pirk^neuro^* in both females and males at Day 28 (Supplementary Fig. 6). Indeed, in females, *pirk^neuro^ imd^neuro^* double knockdown was better than controls (Supplementary Fig. 6).

*Imd* knockdown rescued the changes in total sleep duration (24 h) observed in *pirkglia* and *pirkneuro* at Day 6 (Fig. 5A). This was due to the rescue of defective daytime sleep observed in these flies (Fig. 5B). The significant differences in daytime sleep in both 6- and 28-day-old *pirk^ISC^* flies were also rescued with concomitant knockdown of *imd* (Fig. 5C). Beyond rescue of 24 h and day sleep time, there was recovery of sleep quality *via* alternations to sleep bout duration. At Day 6, *pirkglia imd^glia^* and *pirk^neuro^ imd^neuro^* had sleep bout duration that was statistically indistinguishable from controls (Fig. 5D). Restoration to the quality of daytime sleep observed in glia and neurons were also observed when *imd* was silenced in ISCs. Specifically, duration of each sleep bout at Days 6 and 28 was restored to control levels in *pirkISC imd^ISC^* flies (Fig. 5E).

Night-time sleep defects observed in nervous system-specific *pirk* knockdown were not recovered by concomitant *imd* knockdown. With regards to night-time duration and quality of sleep, the *pirkglia imd^glia^* double mutant phenotype was not significantly different to the *pirk^glia^* or GAL4 control at Day 6 (Supplementary Fig. 8A). Compared to GAL4-only, no changes were seen for *pirk^glia^* at Day 28 and this phenotype was not modified by *imd* knockdown. For *pirk^neuro^*, altered night-time sleep duration and quality were not recovered at Day 6, but there was significant recovery of the reduced frequency of sleep episodes and elevated episode duration at Day 28 (Supplementary Fig. 8B). This suggested rescue of sleep quality (less fragmented sleep), despite no net changes in total night-time sleep duration. Taken together, sleep results with concomitant *pirk* and *imd* knockdown indicated rescue of the *pirk* RNAi phenotypes.

### Age-dependent increase in density and decrease in diversity of the *pirk^ko^* bacteriome

Compared to humans, the gut microbiota of *D. melanogaster* in the laboratory is of low diversity, with *Acetobacter* and *Lactobacillus* spp. dominating the bacteriome.^50-53^ It is thought that *Lactobacillus* spp. dominate in young flies while *Acetobacter* spp. increase in older individuals.^50-53^ Previous studies have shown that both density and diversity of gut bacteria is highly flexible,^52,53^ with a complex set of interactions between gut bacteria and host based on different host diets.^54^

Since *pirk* expression in the gut is microbiota dependent^21^ we explored the density and diversity of gut bacteria in *pirk^ko^* mutants. *Pirk^ko^* flies had significantly more bacterial colonies in their gut than *w^1118^* controls on MRS agar (facilitating detection of *Lactobacilli*) at Day 28 but not Day 6 (Supplementary Fig. 9A). To confirm whether this result was consistent across a wider array of cultivable bacteria, the experiment was repeated using LB agar. This resulted in a significant increase of bacterial colonies again at Day 28 but not Day 6 (Supplementary Fig. 9B). These results indicated an age-dependent increase of gut bacteria density in *pirk^ko^* mutants.

**Figure 6:**
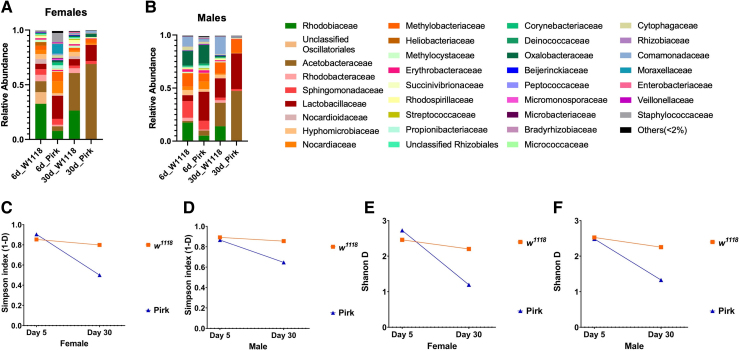
***Pirk^ko^* mutants have reduced gut bacterial diversity.** Next generation sequencing of 16S libraries determined the relative abundance of gut bacterial families observed in 6- and 30-day-old *pirk^ko^* flies and their controls **(A)** Relative abundance in females compared to their controls (*n* = 40/strain). **(B)** Relative abundance in males compared to their controls (*n* = 40/strain). When only the most prominent families were considered (Simpson’s index) an age-dependent reduction in diversity was observed in **(C)** *pirk^ko^* females (0.9135 versus 0.8617 in controls at Day 6; and 0.5048 versus 0.8067 in controls at Day 30) **(D)** *pirk^ko^* males (0.8746 versus 0.9008 in controls at Day 6; and 0.6532 versus 0.8639 in controls at Day 30) both compared to age- and sex-matched controls. When all bacterial families were considered (Shannon-H index) again a significant age-dependent reduction in diversity was observed in **(E)** *pirk^ko^* females (2.897 versus 2.629 in controls at Day 6; to 1.353 versus 2.373 in controls at Day 30) and **(F)** *pirk^ko^* males (2.655 versus 2.691 in controls at Day 6; to 1.489 versus 2.424 in controls at Day 30) both compared to age- and sex-matched controls.

To investigate the effect of *pirk^ko^* on the bacterial composition of the gut we performed high-throughput DNA sequencing of 16s rRNA libraries. We first analysed the relative abundances of bacterial families detected in the guts of both 6-day- and 30-day-old female and male *pirk^ko^* flies compared to their *w^1118^* controls (n = 40 guts/strain). The midgut of 6-day-old *w^1118^* female flies were dominated with Rhodobiaceae (∼32.5%) and Acetobacteraceae (∼10%) and unclassified Oscillatoriales (∼10.5%). However, this dominance was not observed in *pirk^ko^* 6-day-old female flies where we found Lactobacillaceae (∼21%) as the dominant species, along with Nocardiaceae (∼11%) and Moraxellaceae (∼9.2%). The relative abundance of Acetobacteraceae was shown to have severely declined in *pirk* mutants, from 10% in controls to 4% in young female *pirk^ko^* flies. This reduction in Acetobacteraceae correlated with an increase in Lactobacillaceae from 4% in control flies to 21% in 6-day-old *pirk^ko^* female flies (Fig. 6A). In contrast to the 6-day-old female flies, the 30-day-old female flies were dominated by Acetobacteraceae, with 34% in *w^1118^* female flies and 68% in *pirk^ko^* female flies. The guts of older *w^1118^* flies also contained 26% Rhodobiaceae and 6% Lactobacillaceae. However, only two families made up ∼83% of the total bacterial population in the gut of 30-day-old *pirk^ko^* female flies namely, Acetobacteraceae (68%) and Lactobacillaceae (14%). Interestingly, we observed a 17-fold age-dependent increase in the relative abundance of the Acetobacteraceae family in *pirk^ko^* mutant female flies in comparison to a 3.4-fold increase in *w^1118^* female flies (Fig. 6A).

The gut of male 6-day-old *w^1118^* flies was dominated by Rhodobiaceae (17.5%). However, other families such as Sphingomonadaceae (15.6%) and Oxalobacteraceae (13.7%) were also observed. We found that the *w^1118^* male gut microbiome only contained 5.4% Lactobacillaceae and 1.4% Acetobacteraceae. In contrast, *pirk^ko^* male flies, harboured 27% Lactobacillaceae and 4.6% Acetobacteraceae. Other dominant families were Oxalobacteraceae (17.5%) and Sphingomonadaceae (8.3%) (Fig. 6B).

At 30 days, *w^1118^* male gut composition was dominated by Lactobacillaceae (22%) and Acetobacteraceae (18%), along with Comamonadaceae (16.6%) and Rhodobiaceae (14%) while the *pirk^ko^* male microbiome harboured 46% Acetobacteraceae and 33% Lactobacillaceae (Fig. 6B). As in previous studies (Wong *et al.*, 2011; Mistry *et al.*, 2017), we observed an age-dependent increase of Acetobacteraceae in both controls and *pirk^ko^* male flies. However, we observed a 10-fold increase in Acetobacteraceae composition in *pirk^ko^* flies (from 4.6% to 46%) and a slight decrease in Lactobacillaceae (from 27% to 22%) composition. Similarly, we noted a 12-fold increase in Acetobacteraceae composition in *w^1118^* control flies (from 1.4% to 18%) along with a 4-fold increase in Lactobacillaceae abundance (5.4% to 22%).

Analysis by alpha indices showed that in both females (Fig. 6C and E) and males (Fig. 6D and F) the diversity in *w^1118^* over the time course was relatively stable even if relative abundance changed from young to older flies (see above). In contrast, diversity was reduced in *pirk^ko^* mutants in both females (Fig. 6C and E) and males (Fig. 6D and F) with older flies carrying a less diverse intestinal bacteriome. To conclude, our data indicated that *pirk^ko^* flies displayed an age-dependent increase in density of gut bacteria but a corresponding loss of diversity.

We next asked if the *pirk^ko^* microbiota could modify behaviour in control, initially germ-free flies. Our lab has previously demonstrated co-housing as a method of transferring a fly genotype’s microbiome to another fly genotype.^23^ Mistry *et al.*,^23^ co-housed *yw* (control) flies with *pirk;trbd* flies, which had a constitutively active immune response due to the inactivation of two negative regulators of the Imd pathway namely, *pirk* and *trabid.*^23^ In this context, we observed that despite having a different gut bacteria composition at the start, *yw* flies showed similar composition to *pirk;trbd* as early as 14 days of co-housing indicating that the *pirk;trbd* microbiome displaced the *yw* one.^23^ This result illustrated the possibility of a ‘microbiome transfer’ from one fly strain to another.^23^ For the present study, 1-day-old germ-free *yw* flies were cultured for 5 days in food vials used by 6-day-old or 28-day-old *pirk^ko^* flies. Germ-free *yw* controls were cultured in normal vials previously occupied by their 6-day-old or 28-day-old *yw* siblings. We observed a statistically significant decline in total sleep duration of *yw* female flies when housed in 6-day-old *pirk^ko^* food [*P* = 0.0032 (*n* = 32X3)] (Supplementary Fig. 10A). Moreover, a more modest but still significant decline was observed when *yw* females were housed in 28-day-old *pirk^ko^* food for 5 days [*P* = 0.0302 (*n* = 32X3)] (Supplementary Fig. 10A). This correlated with a significant increase in latency to sleep (how long it takes for a fly to fall asleep) during the day at the 6-day time point (Supplementary Fig. 10B) and during the night at the 28-day time point (Supplementary Fig. 10C). In addition, 1-day-old initially germ free male *yw* flies housed in 6-day-old *pirk^ko^* food for 5 days, showed an increase in 24 h sleep (Supplementary Fig. 11A). No changes were observed in total sleep when housed in 28-day-old *pirk^ko^* food (Supplementary Fig. 11A). Nevertheless, as their female siblings, *yw* males displayed an increased latency to sleep in both daytime (Supplementary Fig. 11B) and night-time (Supplementary Fig. 11C) when cultured in *pirk^ko^* food.

Taken together, results on the diversity and density of gut bacteria argue for intestinal bacteria changes in the absence of *pirk* and suggest a causative role for the microbiome in parts of the neurological phenotypes observed related to sleep patterns.

### Axenic conditions only partially rescue the neurological decline of *pirk^ko^* flies

**Figure 7:**
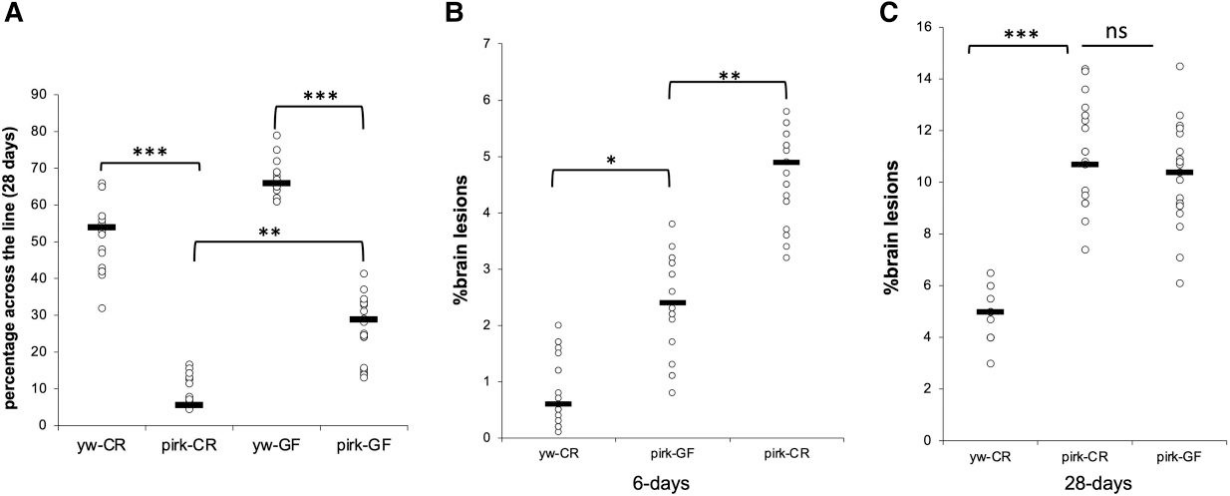
**Axenic conditions rescue early but not late neurodegeneration in *pirk^ko^* mutants. (A)** Germ-free *pirk^ko^* female flies had an increased climbing ability relative to their conventionally reared (CR) siblings but they were significantly slower that GF controls. Each dot represents a single fly (total *n* = 45, three biological repeats) **(B)** Axenic conditions rescued early neurodegeneration in 6-day-old *pirk^ko^* female flies. Each dot represents one fly brain (total *n* = 30, three biological controls) **(C)** However, germ-free conditions did not rescue neurodegeneration in 28-day-old GF *pirk^ko^* female flies, which were statistically indistinguishable from their CR siblings. Each dot represents one fly brain (total *n* = 30, three biological repeats). Analysis with two-way ANOVA and Tukey’s *post hoc* test (*P < 0.05, **P < 0.01, ***P < 0.001).

*Pirk* mutants presented with an age-dependent increase in density but an age-dependent reduction in diversity of gut bacteria. This altered bacteriome influenced the observed neuropathology. At Day 28, axenic (germ-free or GF) *pirk^ko^* flies had an ameliorated locomotion profile compared to their CR siblings while still significantly reduced in comparison to GF or CR *yw* controls (Fig. 7A). Previous studies have shown that loss of *pirk* leads to early brain neurodegeneration.^11^ Quantification of eosin brain staining at Day 6 revealed that GF *pirk^ko^* flies had a significant reduction in brain lesions compared to their CR siblings but still higher than the axenic controls (Fig. 7B). At 28 days however, GF *pirk^ko^* flies had a brain lesion profile that was statistically indistinguishable from their CR siblings (Fig. 7C).

Taken together these results revealed that the gut microbiome partly contributed to the neurological decline of *pirk^ko^* flies. Moreover, gut bacteria influenced early rather than late adulthood as raising *pirk^ko^* germ free, delayed but did not prevent brain lesions from forming later in life indicating a microbiota-independent role for brain immunity in the process.

## Discussion

Human genetics and animal model research have illustrated the correlation between innate immunity in the brain and the pathogenesis of neurodegenerative disorders.^1-5,13,39^ Results from these studies indicate the role of the immune system as an aetiological mechanism influencing not only the pathology of disease but also the basal levels of age-dependent neurodegeneration in the context of healthy ageing. Studies in *Drosophila* suggest that this is an evolutionarily conserved process. Predisposing flies to chronic immune activation by knockouts of Imd negative regulators, overexpressing AMPs in the brain, or dysregulating autophagy (leading to induction of AMP genes), all resulted in age-dependent neurodegeneration.^10-12^ Distinct from all other Imd intracellular negative regulators, *pirk* expression in ISCs is reliant on NF-κB and the presence of gut bacteria.^21^ However, we do not know how loss of *pirk* influences ISC turnover. What we do know from previous studies, is that *pirk* mutants exhibit early neurodegeneration and reduction of lifespan.^11^ The latter phenotype was rescued in axenic conditions, which suggested that neurological decline and loss of neurons in the brain may also be rescued in germ-free flies^11^ Here, our data show that axenic conditions only partially rescued the neurological and neurodegenerative phenotypes of *pirk^ko^* flies. Germ-free *pirk^ko^* flies had a later onset of their neurological phenotypes as indicated by brain histology and locomotion assays. However, transplantation of the *pirk^ko^* microbiome to control flies did not influence climbing ability but only sleep.

Results obtained by cell-specific *pirk* RNAi build a picture on the tissue-specificity of the loss of *pirk*. Silencing (RNAi) of *pirk* in intestinal stem cells (*pirk^ISC^*) resulted in early (Day 6) neurological defects. In contrast, silencing of *pirk* in the nervous system exhibited later neurological defects (Day 28) most prominently in glia (*pirk^glia^*). Our working hypothesis is that changes in gut bacteria caused by the absence of *pirk*, influences neuropathology at early stages of adulthood while chronic immune activity in glia, caused by *pirk* knock down using the pan-glia *GAL4* driver *repo-GAL4,* contributes to neurodegeneration at later stages of adulthood. This corroborates with previous findings that indicate suppression of neurodegeneration when, in the context of chronic Imd activation, NF-κB signalling is blocked in glia.^11^

One limitation in our sleep assays is the potential survivorship bias introduced by using 28-day-old *pirk^ko^* flies. The 28-day time point inherently reflects a population with significant mortality (70% of the cohort has already died, as per Figs 1 and 3). This introduces unavoidable survivorship bias, as only the ‘fittest’ subset of the original population remains. However, as shown in Fig. 2C and F activity levels of these flies appear stable when compared to their younger 6-day-old siblings. Nevertheless, we acknowledge that the physiology of these survivors may differ systematically from younger flies or from controls with longer lifespans (e.g. altered metabolism, compensatory mechanisms or stochastic resilience unrelated to the *pirk* mutation itself). For example, increased/decreased activity/sleep in 28-day-old mutants could reflect either a direct effect of *pirk* or a survival advantage for flies with specific sleep/wake patterns. This is particularly pertinent for the male data, which were shorter lived than females.

In humans, disturbances in sleep patterns have been connected to neurodegeneration.^41-46^ Here, we have measured several parameters to understand sleep patterns in flies predisposed to chronic inflammation. These were day and night-time sleep, number of sleep episodes and time of each episode (more sleep episodes with less time than normal each, means fragmented sleep), as well as latency of sleep (how easy it is to get to sleep). Immune-dependent neurological decline was associated with significant increase of daytime sleep in *pirk^ko^*, *pirk^ISC^ and pirk^glia^* flies. This increase was recovered by concomitant *pirk* and *imd* knockdown and in *pirk^ko^; AttD* mutants. Sleep defects could be a downstream effect of neurological decline or a contributing factor. Meanwhile, as no night-time sleep defects were recovered by *imd* knockdown (Supplementary Fig. 8), this suggests that night-time sleep defects may emanate from the onset of other factors involved in neurological decline, particularly factors in nervous tissue as glia and neurons appear to mediate most of the night-time sleep defects observed. Hence, the contribution of immune dysregulation to defective sleep associated with neurological decline is more prominent in daytime than night-time sleep, suggesting that, in flies at least, daytime sleep defects may be a better biomarker for understanding the role of immune dysregulation in the onset of neurodegeneration.

Silencing *imd* in glia returned total sleep duration, total number of sleep episodes, frequency of sleep episodes and locomotion to wild type levels at Day 28 but not Day 6. Conversely, *imd* knockdown in the gut but not the nervous system recovered neurological defects of *pirk* knockdown at Day 6. Hence, observed neurological decline contributed by overactivation of the Imd pathway early, appeared to originate in the gut. Nevertheless, the neurological phenotype observed at Day 28 cannot be wholly attributed to overactivation of the Imd pathway in the gut: whilst *pirk^ISC^* appeared to contribute widely to the neurological phenotype across the life course, there was no rescue of locomotion by *imd* knockdown in ISCs at Day 28, showing that these defects were independent of gut dysregulation. These results place a temporal element in the contributions of gut and brain towards the sleep changes observed in *pirk^ko^* flies, with the gut mostly contributing early while the effects of the chronic activation of *imd* in the brain being manifested late. One limitation in discussing the role of ISCs in the early onset of neurological effects in *pirk* RNAi is that *esg-GAL4* has also been found to be expressed in neurons, Malpighian tubules and the *corpus allatum* (site of expression of juvenile hormone) in the proventriculus of the gut.^55^ Therefore, RNAi of *pirk* in those sites in addition to the gut, could be causing the phenotype earlier due simply to more widespread depletion.

Nevertheless, the involvement of gut bacteria in the onset of neurodegeneration correlates with the finding that intestinal-specific *pirk* depletion (*pirk^ISC^*) induced earlier onset of neurological decline. This could happen in two (non-mutually exclusive) ways. Either by metabolites derived from commensal gut bacteria passing the blood brain barrier or by gut bacteria capable of breaching an inflamed blood brain barrier. This would correlate with the finding of bacteria in the brains of Alzheimer’s disease patients,^56^ although this has been challenged.^57^ Studies in *Drosophila* models of neurodegeneration have already found a correlation between dysbiotic gut flora and aggravated brain cell death.^25^ An alternative explanation would be the involvement of reactive oxygen species as reactive oxygen species accumulation in the gut has been linked to chronic sleep deprivation in *Drosophila* leading to premature death.^58^ However, the possibility exists that the influence of the microbiota is not derived from the gut, but from the surrounding environment of the flies. More work is needed to distinguish between these scenarios.

Results from a recent study using loss of function mutants, indicate that individual AMPs do not play a role in healthy aging.^49^ In the context of chronic immune activation however, our results provide evidence that AttD is involved in brain cell death, either directly or indirectly. A recent paperhas identified AttD as a non-secreted peptide implicated in immune-induced cell damage caused by Imd activation in Malphigian tubules.^27^ This indicates that AttD can drive cell death elsewhere in the fly. The fact that it is non-secreted however, combined with the fact that *AttD* is expressed in the brain and its knockout recovered brain lesions indicates a cell-autonomous function of AttD associated with the brain. A limitation in this is that the third chromosome of *ΔΑμP-10* also deletes the AMP *drosomycin*. In our hands, the latter is not upregulated in pirk-deficient brains.

Loss of *AttD* suppressed most of the defects which were associated with neurological decline. For example, there was recovery of the defective daytime sleep seen in flies with a neurological phenotype, but there was no recovery of defective night-time sleep. This suggested that when immunity is chronically activated, *AttD* likely regulates sleep during the day but there are other AMPs (or other factors) responsible for night-time sleep regulation. A candidate for this is the sleep-promoting AMP Nemuri.^59^ Whilst chronic expression of *AttD* in *pirk^ko^* mutants curtailed normal lifespan, overexpression of single AMPs, including Drosocin and CecropinA1, showed a correlated increase of lifespan in a microbiota-dependent manner, suggesting a wide breadth to AMP function.^60^

Even though animal models do not represent human neurodegenerative diseases fully (for example in the lack of direct orthologues for the human proteins prone to aggregation in Alzheimer’s disease or Parkinson’s disease), comparative studies of brain development and the innate immune response have demonstrated significant evolutionarily conserved mechanisms between vertebrates and invertebrates. Pirk is *Drosophila-*specific and not present in mammals.^19^ Conceptually however, the dysregulation of innate immunity as aetiology for neurodegeneration is valid in a less complex invertebrate brain even in the absence of tau, β-amyloid or gene-for-gene homology.

## Supplementary Material

fcaf144_Supplementary_Data

## Data Availability

All data presented herein are in the main or supplementary figures of this study.
