# Supplementary material for: Loss of the NF-κB negative regulator Pirk in Drosophila links brain and gut immunity to neurodegeneration
Source: Brain Commun. 2025 Apr 15;7(2):fcaf144. doi: 10.1093/braincomms/fcaf144 (PMC12209854; doi:10.1093/braincomms/fcaf144)
Supplement: fcaf144_Supplementary_Data [file fcaf144_Supplementary_Data.pdf]

SUPPLEMENTARY MATERIAL

Loss of the NF-κB negative regulator Pirk in *Drosophila* links brain and gut immunity to neurodegeneration.

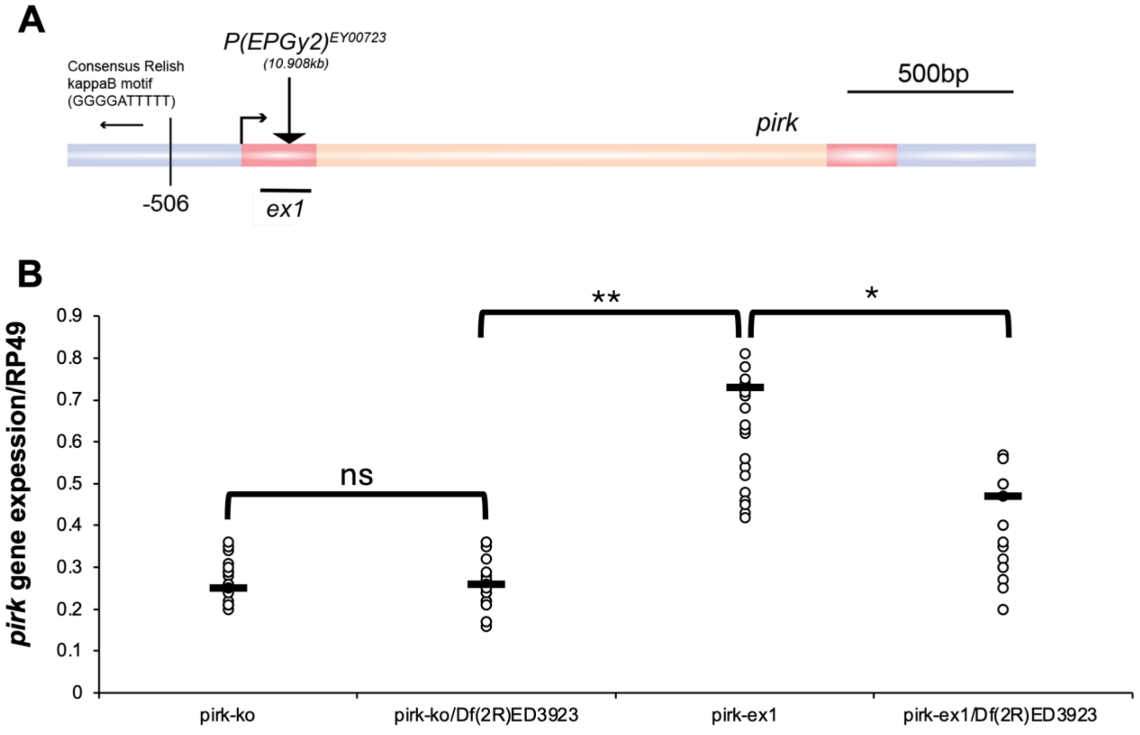

Supplementary Figure 1. The genetics of *pirk.* (A) a schematic diagram representing the position of the P-element insertion in the *pirk* gene (orange), 5' and 3' untranslated regions (red) and NF-κB/Relish binding consensus sequence. **(B)** *Pirk* expression levels in whole flies. Each data point represents relative expression in an individual fly (n=25). Relative *pirk*/ΔC*trp49* ratios of $pirk^{EY0072}$, $pirk^{EY0072}$*/Df(2R)ED3923, the* $pirk^{ex1}$ imprecise excision allele*,* $pirk^{ex1}$*/Df(2R)ED3923.* The control genetic background (*yw*) was set as 1 and the fold differences are indicated. Statistical comparisons were conducted using Kruskal-Wallis test (**p<0.01, *p<0.05). The *Df(2R)ED3923* deficiency uncovers the *pirk* locus.

A

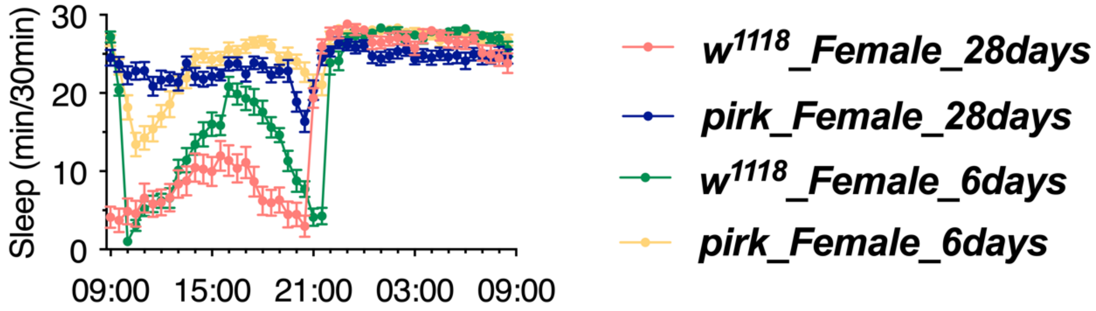

B

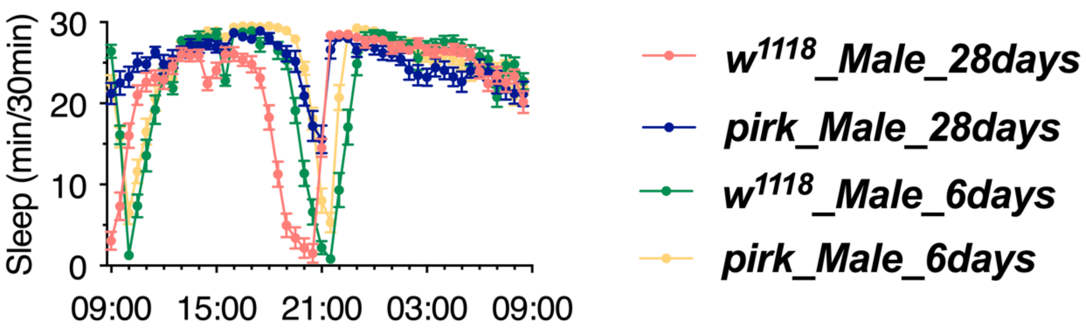

Supplementary Figure 2. Sleep patterns of *pirk*^{ko} in young (6-days old) and older (28-day old) flies.

(**A)** Sleep patterns of *pirk*^{ko} female flies followed a predictable circadian rhythm**.** Total Sleep duration was calculated as total minutes of sleep during a set period. We observed that *pirk*^{ko} had a significant effect on the total sleep duration of female flies at both 6-days and 28-days ($p < 0.0001$, n= 96). The median sleep of 6 and 28-day *pirk* flies were 1226 and 1192 minutes respectively as opposed to 1002 and 806.5 minutes for 6-days and 28-days *w*^{1118} flies respectively.

(B) Sleep patterns of *pirk*^{ko} male flies followed a predictable circadian rhythm. We observed a significant increase in the total duration of sleep in both 6-day ($p = 0.0258$, n=96) and 28-day old ($p<0.0001$, n=96) male *pirk* flies with a median increase in sleep of 62 minutes in younger flies and an increase of 220 minutes in older ones. In both cases, data was evaluated to check if it followed normal distribution by performing a D'Agostino & Pearson test, a 2-way ANOVA

and Tuckey's *post hoc* test was performed, and p values were extracted. N=96 with +SEM error bars. See Fig 2 for details.

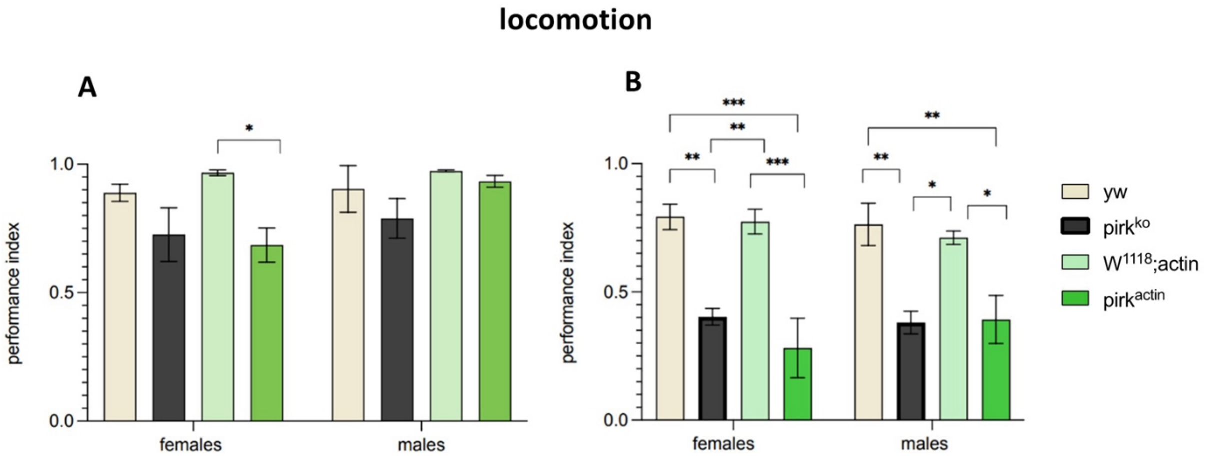

Supplementary Figure 3. Comparing climbing performance of flies with a complete knockout of *pirk* vs. a ubiquitous knockdown (actin).

(A) Locomotion of females and males at day 6

(B) Locomotion of females and males at day 28

Locomotion is represented as a performance index: each bar represents 3 biological replicates, each of which is an average of 3 technical repeats (n=45). Each graph compares *pirk*^{ko} with its yw control and *pirk*^{actin} with its *w*^{1118}; actin-GAL4 control. Values shown (1-way Anova with Tuckey's *post hoc* test) are mean ± SEM (*p<0.05, **p<0.01, ***p<0.001, ****p<0.0001).

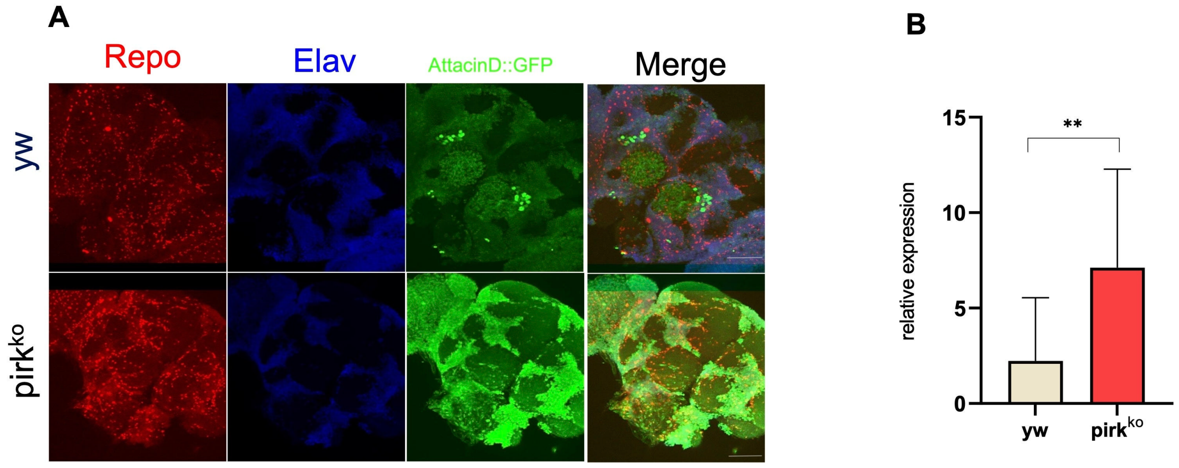

Supplementary Figure 4. *AttD* expression is significantly increased in *pirk^{ko}* flies. **(A)** Brains of 28-day old female *pirk^{ko}* flies showed a significantly increased expression of an *attacinD-GFP* promoter transgene in both neurons (Elav antibody signal) and glia (Repo antibody signal). Scale bar 20µm. **(B)** Quantitative real-time PCR in brains of 28-day old male flies also showed a significant increase in *attD* gene expression. Statistical comparison was conducted using Student's t-test (**p<0.01, n=75).

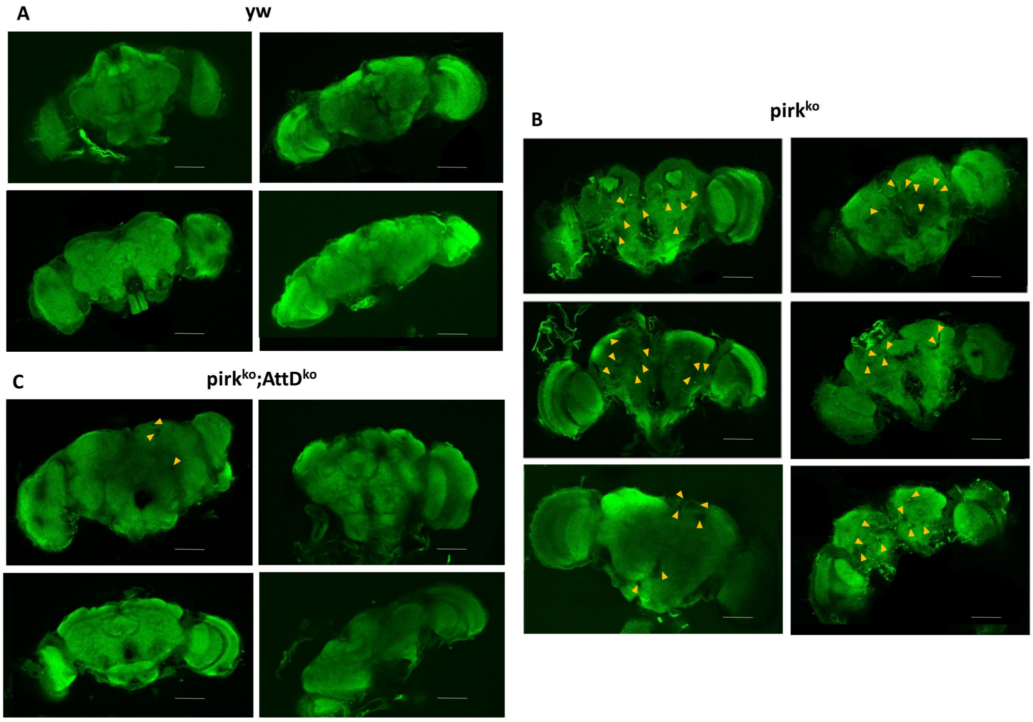

Supplementary Figure 5. Suppression of neurodegeneration in *pirk^{ko};AttD* double knockout. Example regions containing brain lesions are indicated with yellow arrowheads. Brain lesions were identifiable as transient regions lacking stain when moving through the z-stacks (see materials and methods). **a)** *yw* control **b)** *pirk^{ko};AttD* double mutants **c)** *pirk^{ko}*. Transient speckling was seen through most z-stacks in *yw* and *pirk^{ko};AttD* knockouts that could indicate a small degree of neurodegeneration as has been reported elsewhere to be present in healthy ageing11. In contrast, pirkko flies showed additional large regions that were easily identifiable as lesions. Images are of a single z-stack at roughly the midway point of the brain. Shown are two representative brains (20X) per genotype. No significant variation between brains of the same genotype was observed. Samples were blinded [i.e. both the experimenter and the scorer (who were different) did not know the genotypes scored]. Scale bar 20µm. N=35 for each genotype.

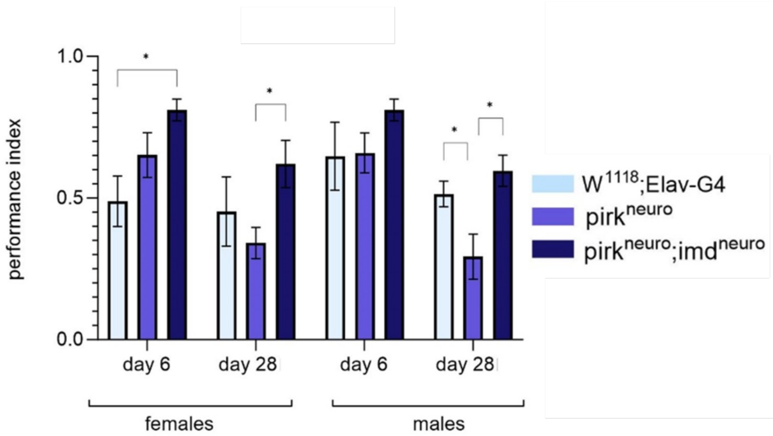

Supplementary Figure 6. Performance of some *pirk*^{neuro} mutants with Imd knockdown exceeded GAL4 controls. *w*^{1118}*; elav-G4, elav-G4; UAS-pirk*^{RNAi}*; UAS-mCherry*^{RNAi} and *elav-G4; UAS-pirk*^{RNAi}*;UAS-imd*^{RNAi} flies are compared in each graph. Locomotion, represented by performance index, is compared separately in female and male flies at day 6 and day 28. Each bar represents 3 biological replicates, each of which is an average of 3 technical repeats (n=45). Analysis with Mann-Whitney test (non-parametric data, independent samples) with Bonferroni correction**.** Values shown are mean ± SEM (*p<0.05).

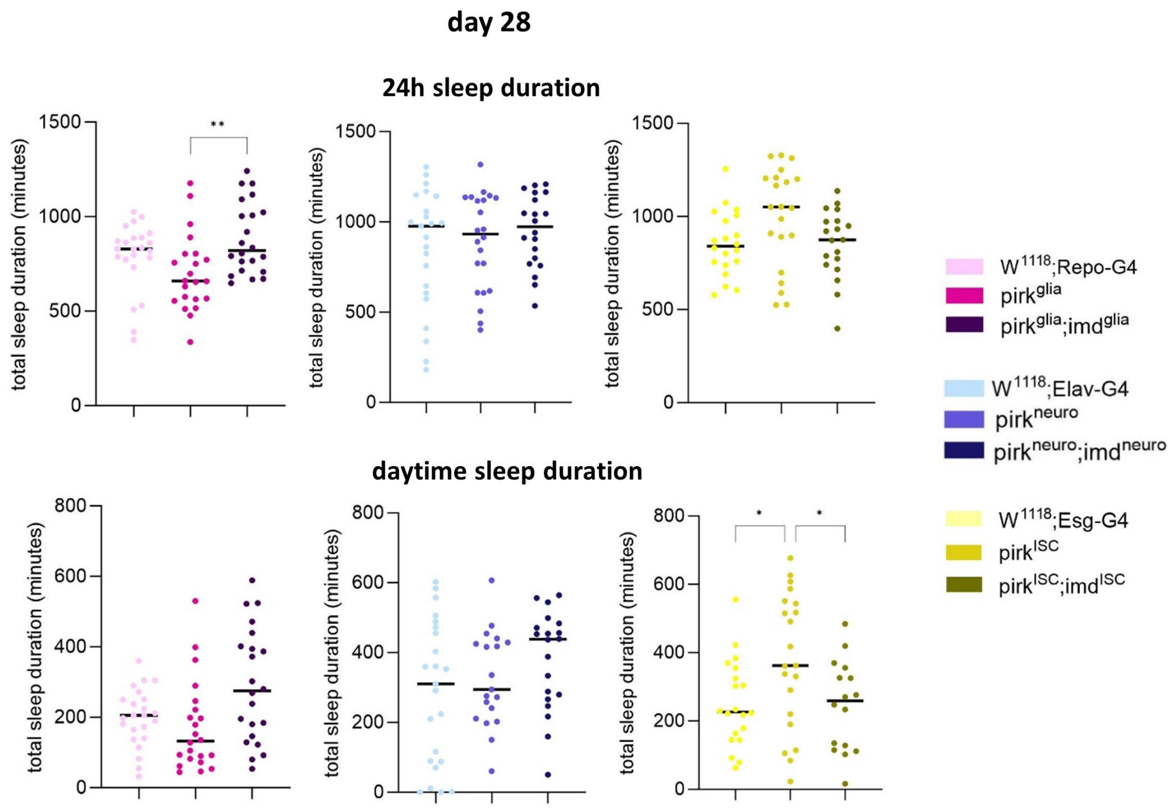

Supplementary Figure 7. Recovery of sleep defects seen in *pirk* knockdowns by *imd* knockdown at day 28. *Pirk^{glia}* flies [*w^{1118};repo-G4*, *UAS-pirk^{RNAi};UAS-mCherry^{RNAi}; repo-G4* and *UAS-pirk^{RNAi};repo-G4/UAS-imd^{RNAi}*] are represented in red, *pirk^{neuro}* flies [*w^{1118};elav-G4, elav-G4;UAS-pirk^{RNAi}; UAS-mCherry^{RNAi} and elav-G4;UAS-pirk^{RNAi};UAS-imd^{RNAi}*] are represented in blue and *pirk^{ISC}* flies [*w^{1118};esg-G4, esg-G4/UAS-pirk^{RNAi}; UAS-mCherry^{RNAi} and esg-G4/UAS-pirk^{RNAi};UAS-imd^{RNAi}*] flies are represented in yellow. Each point denotes a single female fly across 3 biological replicates (n=32X3=96). Analysis with 2-way ANOVA and Tukey's post hoc test (*p<0.05, **p<0.01)

(A) Total sleep duration over 24 hours.

(B) Total daytime sleep duration.

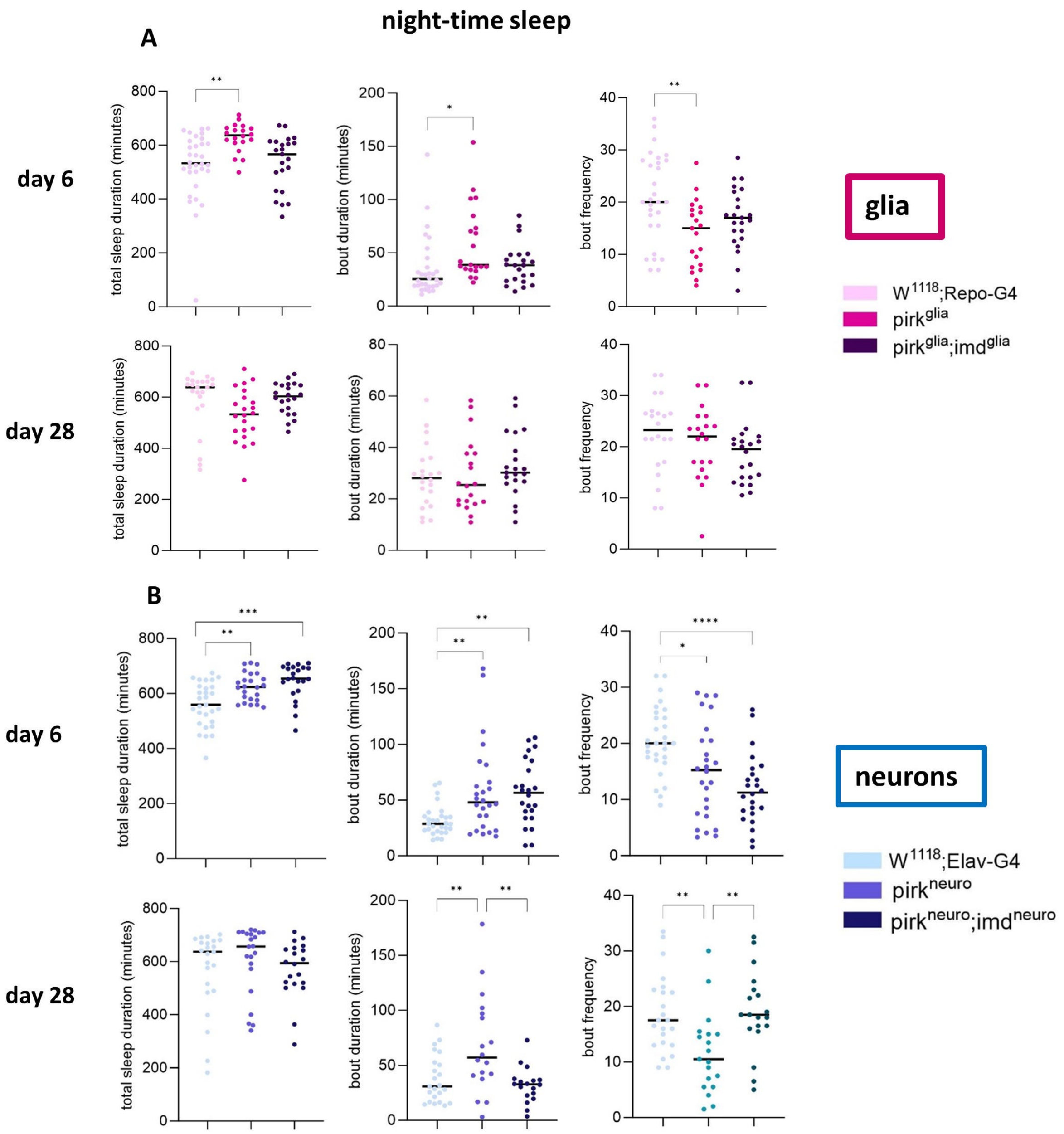

Supplementary Figure 8. No recovery of night-time sleep defects in *pirk*^{glia} and *pirk*^{neuro} mutants by *imd* knockdown. (A) Total duration (left), sleep bout duration (middle) and sleep bout frequency (right) of night-time sleep in *w*^{1118}*;repo-G4, UAS-pirk*^{RNAi}*;repo-G4/UAS-mCherry*^{RNAi} and *UAS-pirk*^{RNAi}*;repo-G4/UAS-imd*^{RNAi} flies at day 6 (above) and day 28 (below). **(B)** Total duration (left), sleep bout duration (middle) and sleep bout frequency (right) of night-time sleep in *w*^{1118}*;elav-G4*, *elav-G4;UAS-pirk*^{RNAi}*/UAS-mCherry*^{RNAi} and *elav-G4;UAS-pirk*^{RNAi}*;UAS-imd*^{RNAi} flies at day 6 (above) and day 28 (below). Each point denotes sleep of a single female fly (3 biological replicates, n=96; *p<0.05, **p<0.01, ***p<0.001, ****p<0.0001, 2-way ANOVA with Tuckey's *post hoc* test).

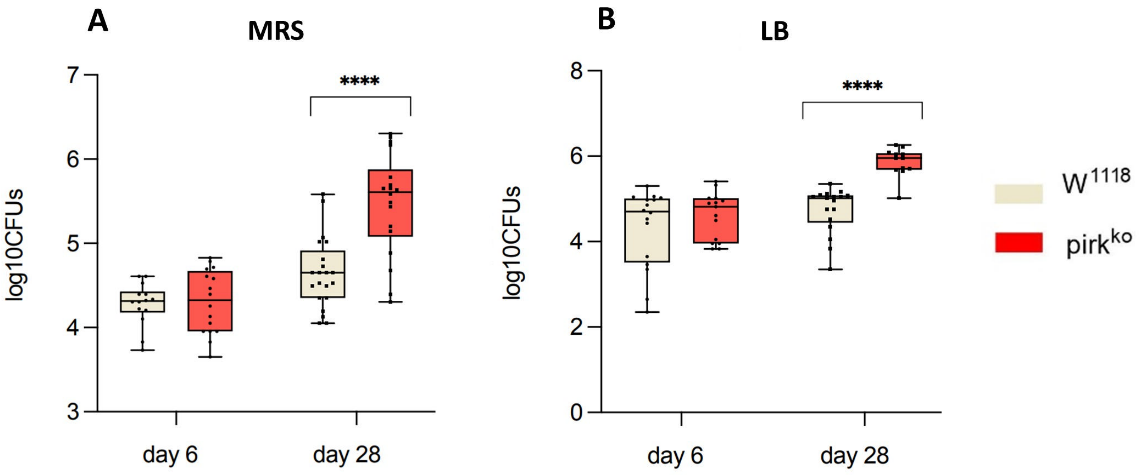

Supplementary Figure 9. *Pirk^ko* mutants had an increased gut bacterial density. Boxplots of log10CFU's of single guts taken from *yw* controls and *pirk^ko* flies at day 6 and day 28. Each point represents a single gut (n=30, 3 biological replicates). Mixed effects analysis. Box plots show all values, with bars indicating min and max values. **(A)** Bacterial density (log10CFUs), on MRS medium. **(B)** Bacterial density (log10CFUs), on LB medium.

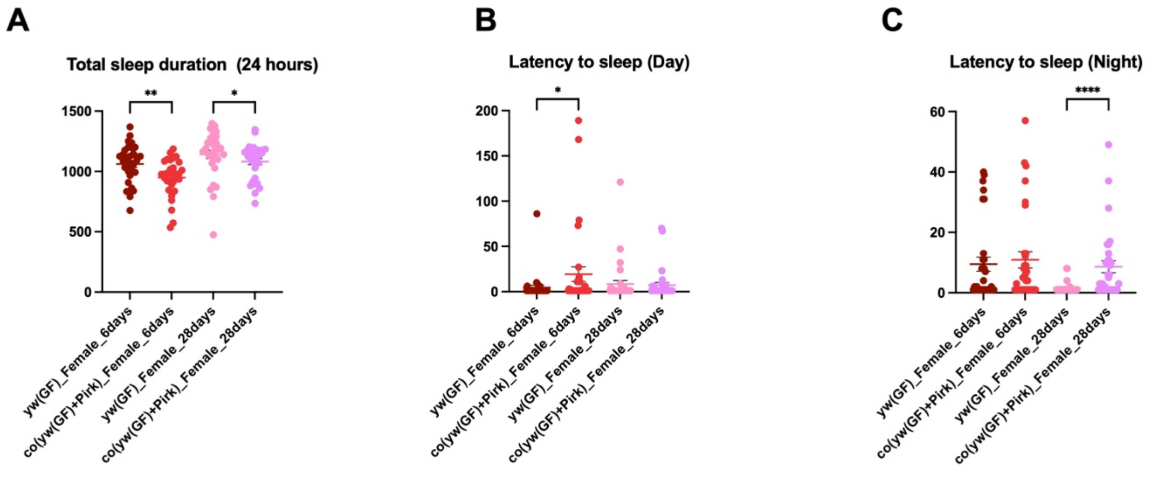

Supplementary Figure 10. Total sleep and sleep latency of control female flies was influenced by the presence of *pirk* microbiota. (A) 24h sleep duration was significantly decreased in both 6 and 28 days. **(B)** This was reflected in an increase in sleep latency in the daytime (6 days) and **(C)** at night (28 days). N=96 (32X3 biological repeats), 2-way ANOVA with Tuckey's *post hoc* test (*p<0.05, **p<0.01, ****p<0.0001).

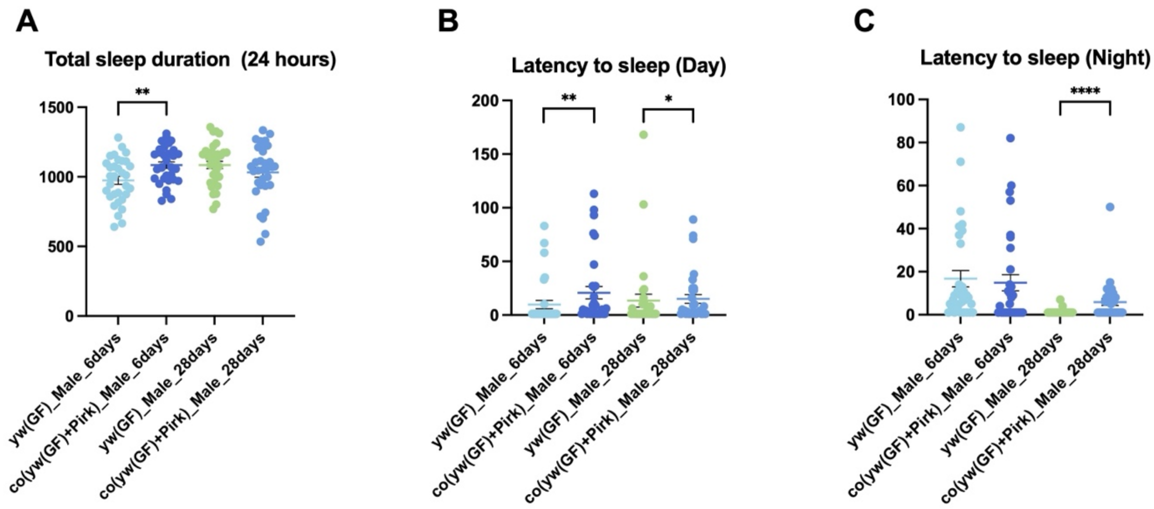

Supplementary Figure 11. Total sleep and sleep latency of control male flies was influenced by the presence of *pirk* microbiota. (A) 24h sleep duration was significantly decreased in both 6 and 28 days. **(B)** This was reflected in an increase in sleep latency in the daytime (6 days) and **(C)** at night (28 days). N=96 (32X3 biological repeats), 2-way ANOVA with Tuckey's *post hoc* test (*p<0.05, **p<0.01, ****p<0.0001).
